# Regulation of matrix reloading by tumor endothelial marker 1 protects against abdominal aortic aneurysm

**DOI:** 10.7150/ijbs.93526

**Published:** 2024-07-02

**Authors:** Yi-Kai Hong, Tsung-Lin Cheng, Chao-Kai Hsu, Fang-Tzu Lee, Bi-Ing Chang, Kuan-Chieh Wang, Lan-Yun Chang, Hua-Lin Wu, Chao-Han Lai

**Affiliations:** 1 Department of Biochemistry and Molecular Biology, College of Medicine, National Cheng Kung University, Tainan, Taiwan.; 2 Department of Dermatology, National Cheng Kung University Hospital, College of Medicine, National Cheng Kung University, Tainan, Taiwan.; 3 International Research Center for Wound Repair and Regeneration (iWRR), National Cheng Kung University, Tainan, Taiwan.; 4 Department of Physiology, College of Medicine, Kaohsiung Medical University, Kaohsiung, Taiwan.; 5 Orthopedic Research Center, College of Medicine, Kaohsiung Medical University Hospital, Kaohsiung Medical University, Kaohsiung, Taiwan.; 6 Regenerative Medicine and Cell Therapy Research Center, Kaohsiung Medical University, Kaohsiung, Taiwan.; 7 College of Professional Studies, National Pingtung University of Science and Technology, Pingtung, Taiwan.; 8 Institute of Clinical Medicine, College of Medicine, National Cheng Kung University, Tainan, Taiwan.; 9 Department of Surgery, National Cheng Kung University Hospital, College of Medicine, National Cheng Kung University, Tainan, Taiwan.; 10 Cardiovascular Research Center, National Cheng Kung University, Tainan, Taiwan.; 11 Department of Medical Laboratory Science and Biotechnology, College of Medicine, National Cheng Kung University, Tainan, Taiwan.; 12 Department of Pharmacy, Chia-Nan University of Pharmacy and Science, Tainan, Taiwan.; 13 Department of Biostatistics, Vanderbilt University Medical Center, Nashville, Tennessee, USA.

**Keywords:** Tumor endothelial marker 1 (TEM1, CD248, endosialin), abdominal aortic aneurysm (AAA), vascular smooth muscle cell, fibroblast, phenotypic change, collagen synthesis

## Abstract

Tumor endothelial marker 1 (TEM1), an activated mesenchymal cell marker, is implicated in tissue remodeling and repair. Herein, we investigated the role and therapeutic implications of TEM1 in abdominal aortic aneurysm (AAA), a potentially life-threatening aortic disease characterized by vascular inflammation and matrix turnover. Characterization of human AAA revealed increased TEM1 expression derived mainly from medial vascular smooth muscle cells (VSMCs) and adventitial fibroblasts. Bioinformatics analysis demonstrated the association between TEM1-expressing VSMCs and fibroblasts and collagen gene expression. Consistently, collagen content and TEM1 expressed by VSMCs and fibroblasts were increased during CaCl_2_-induced AAA formation in mice. *TEM1* silencing in VSMCs and fibroblasts inhibited transforming growth factor-β1-induced phenotypic change, SMAD2 phosphorylation, and *COL1A1* gene expression. Also, *Tem1* deficiency reduced collagen synthesis and exacerbated CaCl_2_-induced AAA formation in mice without disturbing elastin destruction and inflammatory responses. In contrast, rTEM1 promoted phenotypic change and *COL1A1* gene expression through SMAD2 phosphorylation in VSMCs and fibroblasts. Treatment with rTEM1 enhanced collagen synthesis, attenuated elastin fragmentation, and inhibited CaCl_2_-induced and angiotensin II-infused AAA formation. In summary, TEM1 in resident stromal cells regulates collagen synthesis to counteract aortic wall failure during AAA formation. Matrix integrity restored by rTEM1 treatment may hold therapeutic potential against AAA.

## Introduction

Abdominal aortic aneurysm (AAA), a focal weakening and expansion of the abdominal aorta, is a potentially lethal disease in the aged population 1. Open surgery and endovascular repair are the only established approaches for treating AAA. This approach is indicated when the aneurysm reaches 5.5 cm in maximal diameter 2, the threshold at which the risk of rupture substantially increases, given that early elective surgical repair of small AAAs is not beneficial 2, 3. Ultrasonography screening programs are valuable for diagnosing and monitoring aneurysm formation at the early stages of subaneurysmal aortic dilatation and small AAAs 2. In clinical settings, however, no pharmacological therapy has effectively suppressed aneurysm development 2, 3. Physicians remain unable to modify the natural progression of AAA toward eventual rupture.

Exploring molecular mechanisms that drive or halt aortic dilatation may facilitate the development of novel therapeutic strategies for AAA 3-5. The extracellular matrix (ECM) in the aortic wall is composed of matrix proteins that strengthen the vessel walls. Among the primary components of the aortic ECM, elastin provides expandability and recoil properties, and collagen (predominantly collagen type I) is responsible for the tensile strength to withstand the outward forces from high arterial pressure 6, 7. Studies on human AAA specimens and established animal models have shown that transmural inflammation, oxidative stress, and ECM turnover are the hallmark features of AAA development 3, 4, 7, 8. Transmural inflammation, including inflammatory cell (e.g., macrophages) accumulation and cytokine elaboration (e.g., tumor necrosis factor-α [TNF-α], interleukin-6 [IL-6], and monocyte chemoattractant protein-1 [MCP-1]), is apparent in human and mouse AAA specimens. Oxidative stress may be increased and, in turn, contribute to inflammation that may exacerbate the weakness of the aortic wall. Extracellular matrix degradation, as evidenced by proteinase (e.g., matrix metalloproteinase [MMP]) release and elastin fragmentation, is another major part of AAA. Elastin fibers are degraded, whereas collagen content is increased in AAA 6, 9. Collagen synthesis is increased during early aneurysm formation, suggesting a repair process 8, 9. Later, collagen degradation may exceed its synthesis, contributing to aneurysm progression and rupture. Reduced collagen content weakens the aortic wall and exacerbates the aneurysm phenotype in mice 7, 10-12. In this context, stabilization of matrix protein integrity may be a potential therapeutic strategy that confers protection against AAA.

Tumor endothelial marker 1 (TEM1; also known as endosialin or CD248), which is a transmembrane glycoprotein of the C-type lectin-like domain group 14 family 13, 14, was initially identified as a cell surface receptor of the tumor endothelium 15. Later, high-resolution morphological images showed that TEM1 is derived from activated mesenchymal cells (e.g., perivascular pericytes, vascular smooth muscle cells [VSMCs], and interstitial fibroblasts) but not endothelial cells 16-18. TEM1 comprises 6 domains: an N-terminal lectin-like domain, followed by a sushi-like domain, three epidermal growth factor-like repeats, a mucin-like domain, a transmembrane domain, and a cytoplasmic domain 15. TEM1 functions during embryonic development but is barely detected in healthy adults 18. As a tumor vascular marker, TEM1 interacts ECM proteins (e.g., multimerin-2 and fibronectin) and mediates cell adhesion and migration to support tumor progression and invasion 19-21, and these functions have diagnostic and therapeutic importance 22. Besides, our recent study has shown that TEM1 in macrophages contributes to sepsis via its lectin-like domain 23. Implicating a role for TEM1 in tissue remodeling and repair, studies have shown that *Tem1*-deficient mice are protected from hepatic and renal fibrosis 24-26, in line with our observation that TEM1 deletion decelerates wound healing 27.

In studies presented here, we attempted to gain insight into the function of TEM1 in AAA. We have investigated the hypothesis that TEM1 might regulate matrix protein restoration in the aortic wall, thereby protecting against AAA formation. The expression of TEM1 was evaluated in human and experimental mouse AAA formation. Whether loss of TEM1 might affect the development of AAA was evaluated using transgenic TEM1-deficient mice. Subsequently, soluble recombinant TEM1 (rTEM1) containing all the extracellular domains (i.e., from the N-terminal lectin-like domain to the mucin-like domain) was prepared to ascertain its effects in AAA models. These results may provide insights into the role of TEM1 in AAA.

## Results

### TEM1 is highly expressed in human AAA and is localized to medial VSMCs and adventitial fibroblasts

**Figure 1:**
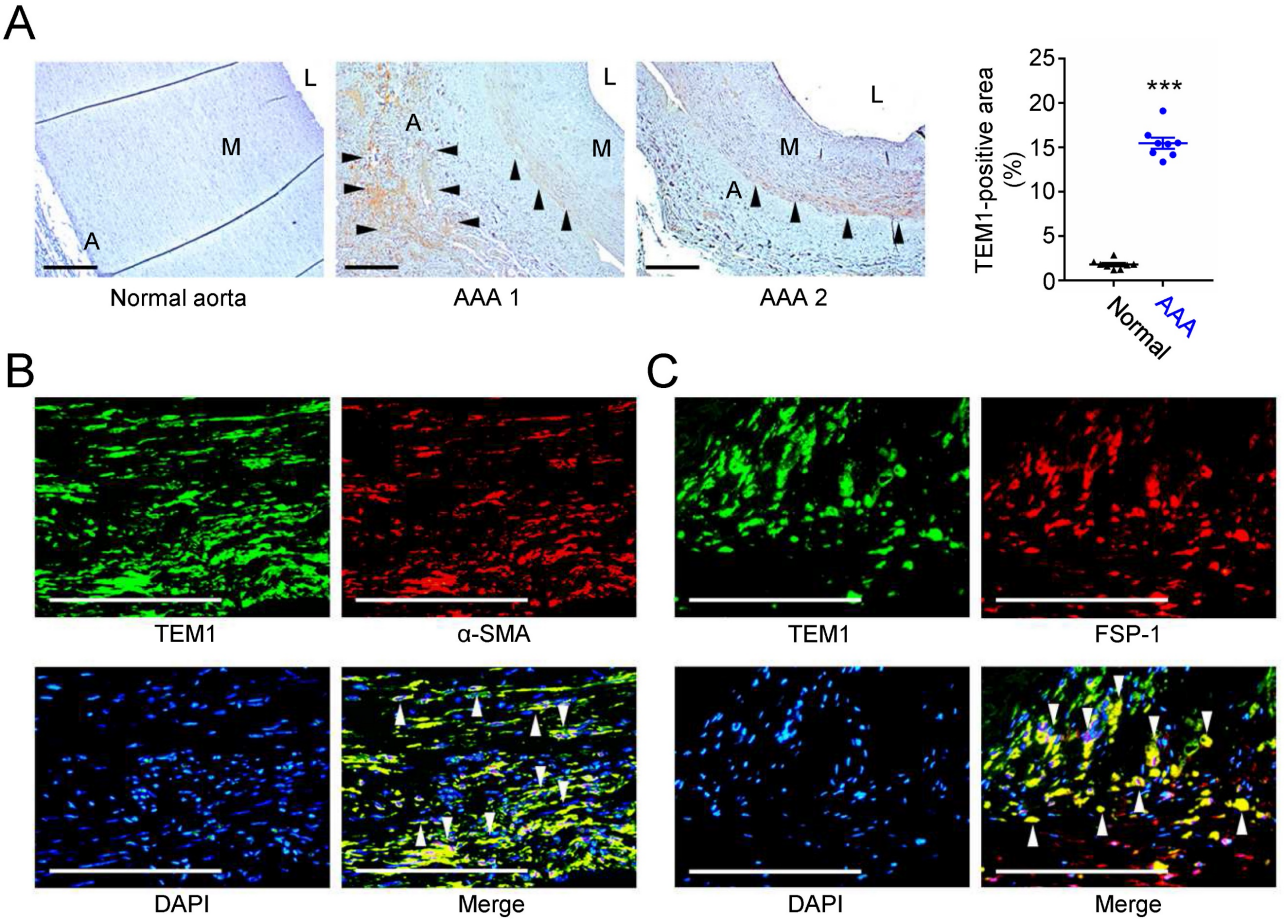
** Increased TEM1 in human AAAs. (A)** Representative microscopic photos of TEM1 expression in the human normal aortas and AAAs and quantification of TEM1-positive area (n=8). Black arrowheads indicate positive TEM1 staining. ***p<0.0001 vs. normal aorta. **(B, C)** Double-immunofluorescent staining for TEM1 with α-SMA (B) and FSP-1 (C), a marker of fibroblasts, in human AAA. White arrowheads indicate overlap of positive staining. (A indicates adventitia. L indicates lumen. M indicates media. Scale bars represent 100 μm. Data are represented as mean values ± SEM.)

First, we examined the expression of TEM1 in human AAA samples. Immunohistochemical staining showed that TEM1 was mainly expressed in the adventitial and medial layers in human AAAs but was scarcely detected in the normal aorta (**Fig. 1A**). Quantitative analysis revealed that TEM1-positive area was significantly increased in AAA. Double immunofluorescence staining revealed that in AAA specimens, TEM1 was expressed in α-smooth muscle actin (α-SMA)-positive VSMCs (**Fig. 1B**) and fibroblast-specific protein-1 (FSP-1)-positive fibroblasts (**Fig. 1C**). These findings implicated a role for TEM1 in the development of human AAA.

### TEM1-expressing VSMCs and fibroblasts are linked to TGF-β signaling and collagen gene expression

**Figure 2:**
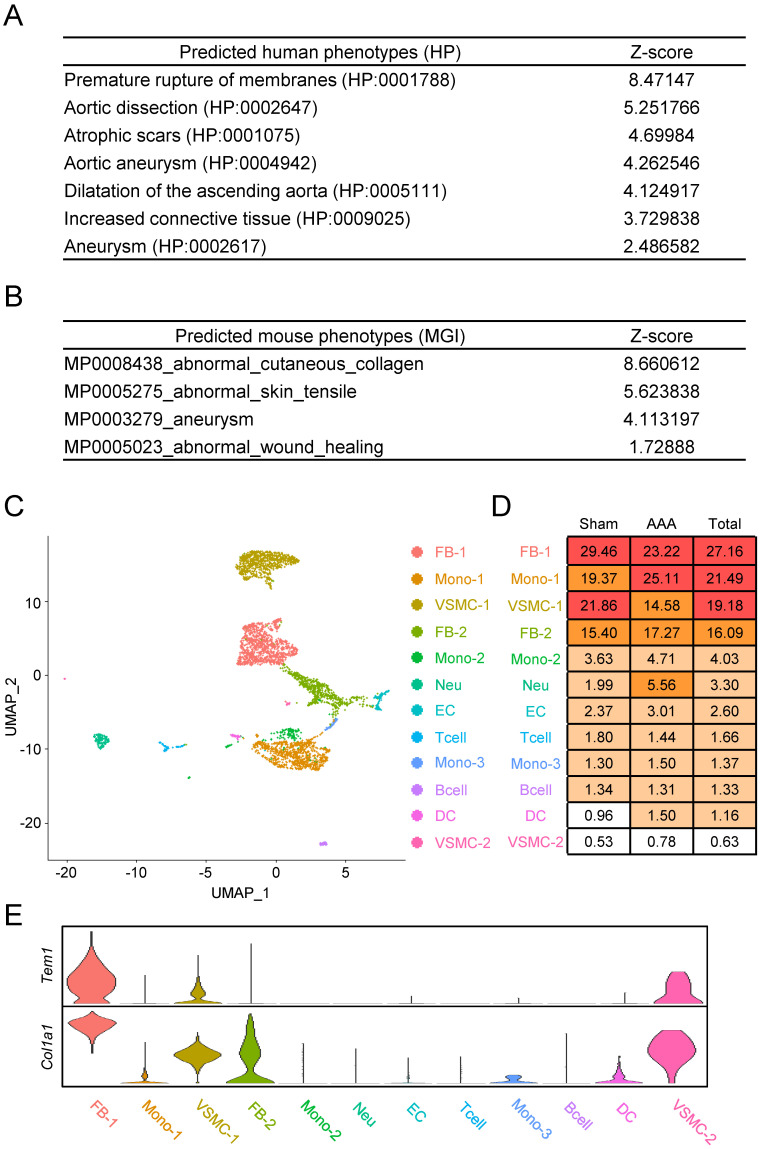
***Tem1* gene expression is linked to VSMCs and fibroblasts in mouse AAA at the single-cell resolution. (A)** Predicted disease phenotypes in humans.** (B)** Predicted disease phenotypes in mice. **(C)** Cellular heterogeneity with 12 distinct cell clusters in mouse AAA. **(D)** The percentage of each cell type are listed. **(E)** The violin plot reveals the relative gene expression of *Tem1* and *Col1a1* in each cell population. (FB, fibroblast; Mono, monocyte; VSMC, vascular smooth muscle cell; Neu, neutrophil; EC, endothelial cell; Tcell, T cell; Bcell, B cell; DC, dendritic cell.)

We next sought to clarify how TEM1 is involved in AAA pathobiology. We leveraged the vast number of publicly available RNA sequencing datasets that are accessible in the ARCHS4 platform 28 to infer the biological and molecular ontologies related to TEM1 in the context of AAA. Gene ontologies (GO) for collagen fibril organization, ECM organization, and smooth muscle tissue development were enriched for *TEM1* gene (**Fig. S1A**).* TEM1* gene was also associated with ECM-receptor interactions and TGF-β signaling in the Kyoto Encyclopedia of Genes and Genomes (KEGG) pathway map (**Fig. S1B**). Notably, predicted disease phenotypes in humans (**Fig. 2A**) and mice (**Fig. 2B**) revealed that *TEM1/Tem1* gene was linked to aortic diseases (e.g., dissection and aneurysm) and tissue fibrosis (e.g., scar and collagen).

Next, we identified specific cell types and interactions involving *Tem1* in the aortic wall using single-cell RNA sequencing (scRNA-seq) of an early-stage CaCl_2_-induced AAA model in mice 29. A total of 4,366 filtered single cells were used for unsupervised clustering, which yielded 12 distinct cell populations (**Fig. 2C, 2D, S2**). Based on these data, we found that *Tem1* and *Col1a1* gene expression was prominent in VSMCs and fibroblasts (**Fig. 2E**).

TGF-β signaling protects against AAA formation 30, 31 and stimulates collagen synthesis in the vasculature 32. Network centrality analysis of the inferred TGF-β signaling network showed that monocytes, dendritic cells, and T cells primarily served as sources, while VSMCs, fibroblasts, and monocytes were the primary targets (**Fig. S3A**). This analysis underscored the autocrine and paracrine effects of TGF-β signaling among these cell types in AAA. Visualizing these interactions in two-dimensional space showed that VSMCs, fibroblasts, and monocytes exhibited robust incoming and outgoing interaction strengths (**Fig. S3B**). Several measures in weighted-directed networks, including out-degree, in-degree, flow betweenness, and information centrality, have been used to respectively identify dominant senders, receivers, mediators, and influencers for intercellular communications 33.

In the network centrality scores (**Fig. S3C**), monocytes were the most active sender, receiver, and mediator cell type, suggesting that monocytes were signal sources and receivers as well as gatekeepers to regulate communication flow between cell groups. In addition, fibroblasts, monocytes, and VSMCs were the dominant influencer cell types, suggesting that those cells had greater control over the information flow. Among the recognized ligand-receptor pairs, TGF-β signaling in AAA was dominated by the *Tgfb1* ligand and multimeric *Tgfbr1/Tgfbr2* receptors (**Fig. S3D**). In summary, data from single-cell profiles suggested that VSMCs and fibroblasts, the cell types expressing TEM1 in human AAA, might participate in TGF-β signaling and collagen gene expression during AAA formation.

### TEM1 expression and collagen production are increased during AAA formation in mice

**Figure 3:**
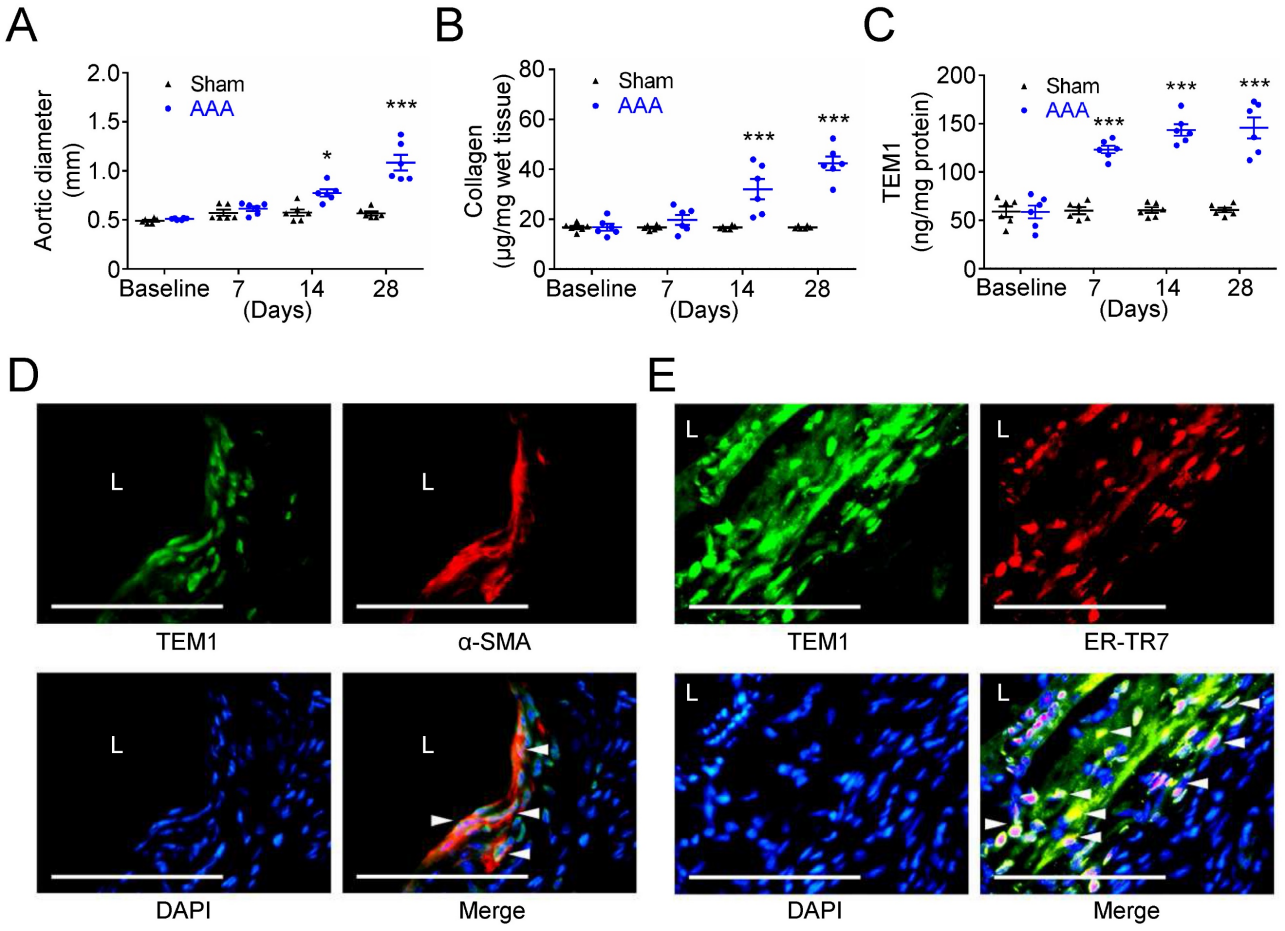
** Increased TEM1 during experimental mouse AAA formation. (A)** Aortic diameter change during mouse AAA formation (n=8 per time point). *p=0.0122 vs. sham. ***p<0.0001 vs. sham. **(B)** Collagen content (n=6 per time point). ***p<0.0001 vs. sham. **(C)** Tissue concentration of TEM1 (n=6 per time point). ***p<0.0001 vs. sham. **(D, E)** Double-immunofluorescent staining for TEM1 with α-SMA (D) and ER-TR7 (E), a marker of fibroblasts, in mouse AAA. White arrowheads indicate overlap of positive staining. (L indicates lumen. Scale bars represent 50 μm. Data are represented as mean values ± SEM.)

Subsequently, we examined TEM1 expression and collagen content in experimental mouse AAA formation that serves as a platform to help recognize the sequence of biological events during AAA development. While the aortic diameter of sham-operated mice was almost unchanged at 28 days, a significant aortic dilatation was observed at 14 days, with further dilatation at 28 days after CaCl_2_ injury (**Fig. 3A**). The aortic samples were analyzed to determine the time-course of collagen levels and TEM1 during AAA formation. Collagen remained constant in sham-operated mice throughout the 28 days. However, collagen was significantly increased at day 14 and day 28 during AAA formation (**Fig. 3B**). Notably, TEM1 was markedly elevated since day 7 (**Fig. 3C**), earlier than the timing of significant aortic dilatation and collagen deposition (14 days; **Fig. 3A & 3B**), and was persistently high during AAA formation. In addition, correlation analysis revealed a moderate positive correlation between aortic diameters and TEM1 (Spearman correlation coefficient=0.5607; **Fig. S4A**) and a strong positive correlation between collagen content and TEM1 (Spearman correlation coefficient=0.7162; **Fig. S4B**). Immunostaining of AAA samples on day 28 showed that TEM1 was predominantly localized to VSMCs positive for α-SMA (**Fig. 3D**) and fibroblasts positive for ER-TR7, a marker of mouse fibroblasts (**Fig. 3E**), compatible with the findings in human specimens and bioinformatics analysis. These results suggested that collagen content and TEM1 derived from resident stromal cells in the aortic wall were increased during CaCl_2_-induced AAA formation.

### TEM1 regulates phenotypic change and collagen gene expression in VSMCs and fibroblasts *in vitro*

**Figure 4:**
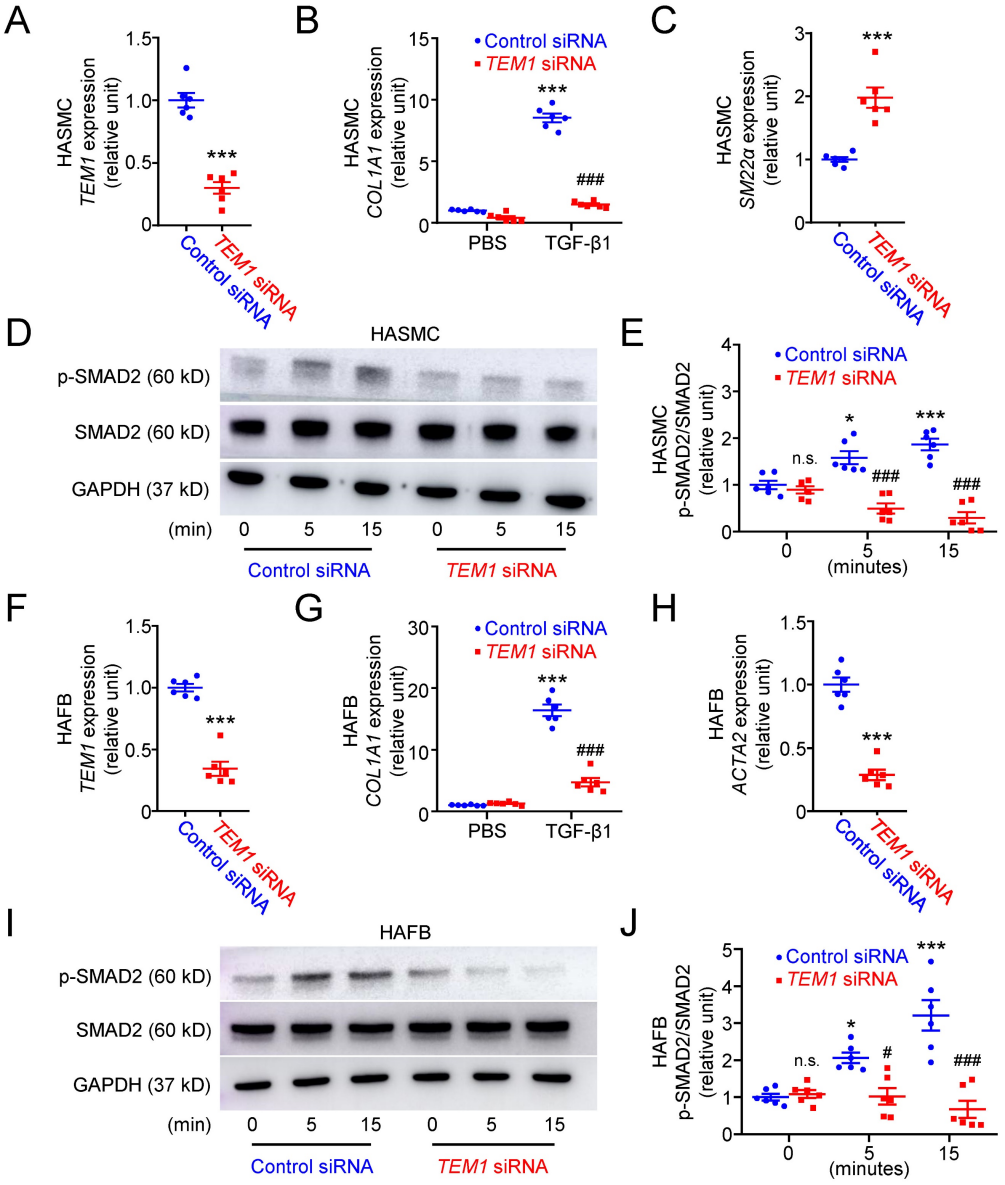
***TEM1* silencing inhibits TGF-β1-induced phenotypic change and collagen synthesis in HASMCs and HAFBs.** HASMCs (A-E) and HAFBs (F-J), transfected with control or *TEM1* siRNA, were treated with 10 ng/ml TGF-β1.** (A)** *TEM1* gene expression (n=6). ***p<0.0001 vs. control siRNA. **(B)** *COL1A1* gene expression levels at 1 day (n=6). ***p<0.0001 vs. PBS, control siRNA; ^###^p<0.0001 vs. TGF-β1, control siRNA. **(C)** *SM22α* gene expression levels at 1 day (n=6). ***p<0.0001 vs. control siRNA. **(D)** Representative western blot analysis of p-SMAD2 and SMAD2 levels at indicated times. **(E)** Quantification of p-SMAD2/SMAD2 levels (n=6). n.s. p=0.9839, *p=0.0362, ***p<0.0001 vs. 0 min, control siRNA. ^###^p<0.0001 vs. 5 or 15 min, control siRNA. **(F)** *TEM1* gene expression (n=6). ***p<0.0001 vs. control siRNA. **(G)** *COL1A1* gene expression levels at 1 day (n=6). ***p<0.0001 vs. PBS, control siRNA; ^###^p<0.0001 vs. TGF-β1, control siRNA. **(H)** *ACTA2* gene expression levels at 1 day (n=6). ***p<0.0001 vs. control siRNA. **(I)** Representative western blot analysis of p-SMAD2 and SMAD2 levels at indicated times. **(J)** Quantification of p-SMAD2/SMAD2 levels (n=6). n.s. p>0.9999, *p=0.0278, ***p<0.0001 vs 0 min, control siRNA. ^#^p=0.0323 vs. 5 min, control siRNA. ^###^p <0.0001 vs. 15 min, control siRNA. (Data are represented as mean values ± SEM).

Based on the results of immunohistochemical and single-cell analyses, we further investigated the function of TEM1 using human aortic smooth muscle cells (HASMCs) transfected with siRNA specifically targeting *TEM1* (**Fig. 4A**).* TEM1* silencing effectively reduced TEM1 protein expression (**Fig. S5A & S5B**) and secretion (**Fig. S5C**) in HASMCs. Transforming growth factor-β1 (TGF-β1) stimulated *COL1A1* expression in HASMCs, and *TEM1* knockdown inhibited TGF-β1-induced* COL1A1* expression (**Fig. 4B**). Additionally, *TEM1* knockdown enhanced the gene expression of the contractile phenotype marker* SM22α* in TGF-β1-treated HASMCs (**Fig. 4C**). Consistently, *TEM1* knockdown increased SM22α protein expression (**Fig. S5D & S5E**), suggesting that TEM1 regulated VSMC switch toward the synthetic phenotype. Time-course analysis of intracellular signaling revealed that TGF-β1 treatment resulted in increased phosphorylation of SMAD2 in HASMCs at 5 and 15 minutes (**Fig. 4D & 4E**). *TEM1* knockdown did not affect the baseline level of SMAD2 phosphorylation in HASMCs but significantly attenuated those at 5 and 15 minutes.

The effects of *TEM1* knockdown (**Fig. 4F**) on TGF-β1-stimulated human aortic fibroblasts (HAFBs) were evaluated. *TEM1* silencing effectively reduced TEM1 protein expression (**Fig. S5F & S5G**) and secretion (**Fig. S5H**) in HAFBs. TGF-β1 stimulated *COL1A1* expression in HAFBs, and* TEM1* knockdown inhibited TGF-β1-stimulated *COL1A1* expression (**Fig. 4G**). *TEM1* knockdown inhibited the expression of the myofibroblast marker* ATAC2* in TGF-β1-treated HAFBs, suggesting that TEM1 regulated TGF-β1-induced fibroblast differentiation into myofibroblasts (**Fig. 4H**). Similar to the analysis of HASMCs, SMAD2 phosphorylation was increased in TGF-β1-treated HAFBs at 5 and 15 minutes (**Fig. 4I & 4J**). Although SMAD2 phosphorylation at baseline was not affected significantly by *TEM1* knockdown, SMAD2 phosphorylation was inhibited by* TEM1* knockdown at 5 and 15 minutes. These findings suggested that TEM1 in VSMCs and fibroblasts mediated TGF-β1-induced phenotypic change, collagen synthesis, and SMAD2 activation.

### *Tem1* deficiency exacerbates aortic dilatation by reducing collagen synthesis in mice

**Figure 5:**
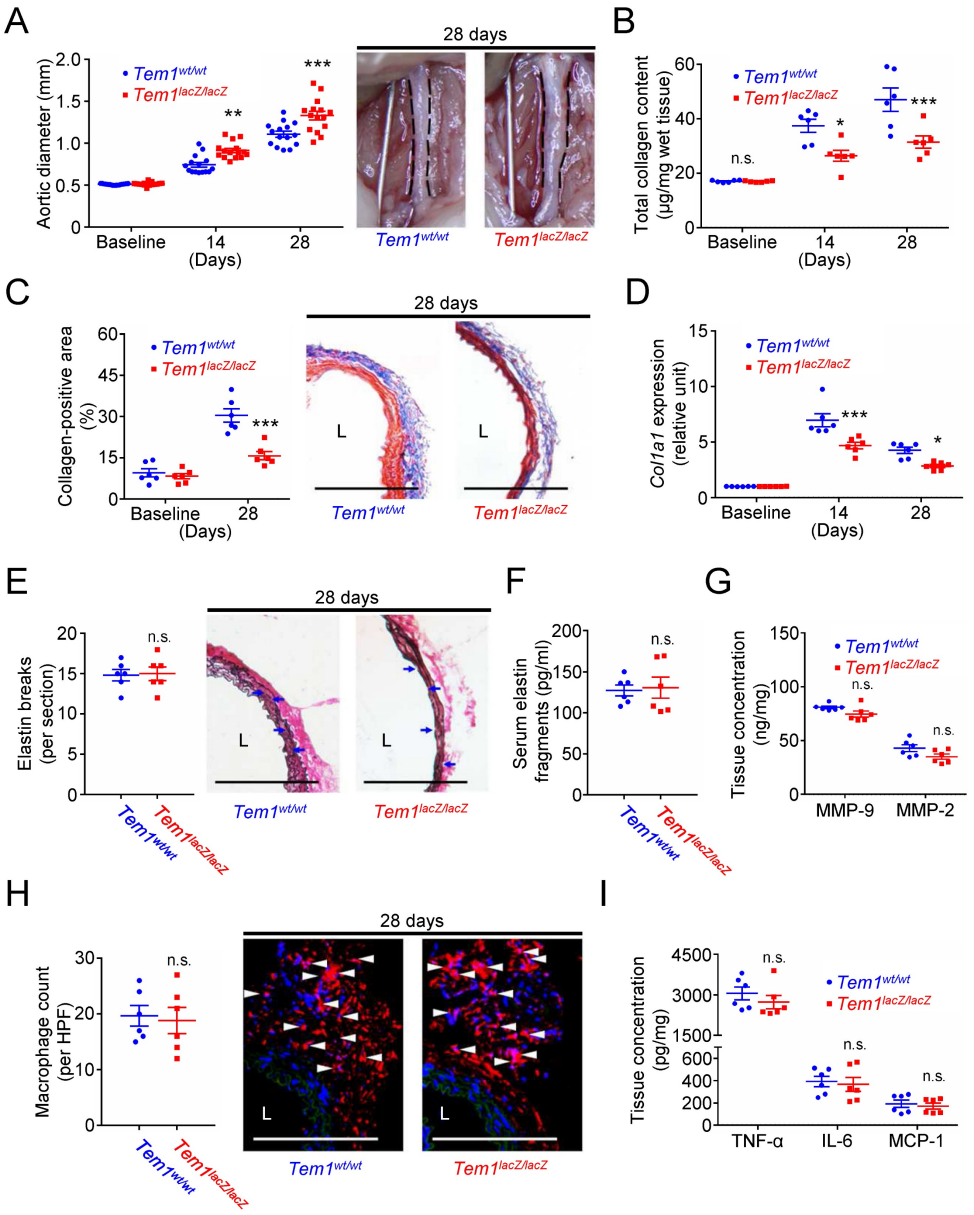
***Tem1* deficiency aggravates experimental aortic dilatation and reduces collagen deposition without interfering with vascular inflammation and proteolysis. (A)** Aortic diameter change (n=15 per time point) and representative microscopic photos of the infrarenal aorta. n.s. p>0.9999, **p=0.0018, ***p<0.0001 vs. *Tem1^wt/wt^*. **(B)** Total collagen content (n=6 per time point). n.s. p>0.9999, *p=0.0360, ***p=0.0009 vs. *Tem1^wt/wt^*. **(C)** Collagen-positive area (n=6 per time point) and representative microscopic images of Masson's trichrome staining. ***p<0.0001 vs. *Tem1^wt/wt^*. **(D)** *Col1a1* gene expression (n=6 per time point). ***p=0.0001 and *p=0.0301 vs. *Tem1^wt/wt^*. **(E)** Elastin break counts (n=6) and representative microscopic images of VVG staining. Blue arrows indicate disrupted elastic lamella. n.s. p=0.8834 vs. *Tem1^wt/wt^*. **(F)** Circulating elastin fragments at 28 days (n=6). n.s. p=0.8250 vs. *Tem1^wt/wt^*. **(G)** Tissue concentration of MMP-9 and MMP-2 at 28 days (n=6). For MMP-9 and MMP-2, n.s. p=0.0783 and n.s. p=0.0743 vs. *Tem1^wt/wt^*, respectively. **(H)** MOMA-2-positive macrophage numbers and representative microscopic images of MOMA-2 staining (with DAPI). White arrowheads indicate MOMA-2-positive, DAPI-stained macrophages. n.s. p=0.7861 vs. *Tem1^wt/wt^*. **(I)** Tissue concentration of TNF-α, IL-6, and MCP-1 at 28 days (n=6). For TNF-α, IL-6, and MCP-1, n.s. p=0.3707, n.s. p=0.7425, and n.s. p=0.6327 vs. *Tem1^wt/wt^*, respectively. (L indicates lumen. All scale bars represent 100 μm. Data are represented as mean values ± SEM).

Collagen is one of the dominant matrix proteins that provide the tensile strength and maintain the structural integrity of the aortic wall 6, 7. Subsequently, transgenic *Tem1*-deficient mice (*Tem1^lacZ/lacZ^* mice) and their wild-type controls (*Tem1^wt/wt^* mice) were prepared to investigate whether TEM1 might regulate collagen synthesis and aortic dilatation* in vivo*. During the development of AAA, the baseline and body weights at 14 and 28 days were not different in *Tem1^lacZ/lacZ^* mice and *Tem1^wt/wt^* mice (**Fig. S6A**). The baseline aortic diameter was not affected by *Tem1* deficiency (**Fig. 5A**). The aortic diameter was 0.74 ± 0.03 mm in *Tem1^wt/wt^* mice and 0.91 ± 0.03 mm in *Tem1^lacZ/lacZ^* mice at 14 days (p=0.0018, 2-way ANOVA followed by Bonferroni correction). In addition, the aortic diameter was 1.11 ± 0.04 mm in *Tem1^wt/wt^* mice and 1.30 ± 0.05 mm in *Tem1^lacZ/lacZ^* mice at 28 days (p<0.0001), suggesting that *Tem1* deficiency exacerbated aortic dilatation. The systolic blood pressure measured at 28 days was not different in *Tem1^lacZ/lacZ^* mice and *Tem1^wt/wt^* mice (**Fig. S6B**). Analysis of aortic samples showed that baseline collagen content was not different between *Tem1^lacZ/lacZ^* mice and *Tem1^wt/wt^* mice. However, collagen content was lower in *Tem1^lacZ/lacZ^* mice than in *Tem1^wt/wt^* mice at 14 and 28 days (**Fig. 5B**), consistent with the collagen-positive area at 28 days shown by Masson's trichrome staining (**Fig. 5C**). Picrosirius red staining, a commonly used histological technique to visualize collagen, showed that collagen content in *Tem1^lacZ/lacZ^* mice was loosely packed and poorly organized compared with that in *Tem1^wt/wt^* mice (**Fig. S6C**). Also, the expression of *Col1a1* was reduced in *Tem1^lacZ/lacZ^* mice compared with *Tem1^wt/wt^* mice at 14 and 28 days (**Fig. 5D**). Atomic force microscopy (AFM) indentation measurements showed that aortic stiffness was lower in *Tem1^lacZ/lacZ^* mice than in *Tem1^wt/wt^* mice (**Fig. S6D**). These findings suggested that *Tem1* deficiency aggravated aortic dilatation due at least partly to impaired collagen synthesis.

ECM turnover, inflammation, and oxidative stress are essential pathological characteristics of AAA development 3, 4, 7, 8. Whether *Tem1* deficiency affected the prominent features of AAA, including proteolytic degradation of ECM (e.g., proteinase release and elastin fragmentation), transmural inflammation (e.g., macrophage accumulation and cytokine elaboration), and accumulation of oxidative stress 3, 4, 8, was examined. Verhoeff Van Gieson (VVG) staining demonstrated that the number of elastin breaks was not different between *Tem1^wt/wt^* and *Tem1^lacZ/lacZ^* mice (**Fig. 5E**). The circulating level of elastin fragments was not different between *Tem1^wt/wt^* and *Tem1^lacZ/lacZ^* mice (**Fig. 5F**). Concentrations of matrix-degrading proteinases in the aortic wall, including MMP-9, MMP-2 (**Fig. 5G**), MMP-1 (collagenase-1), MMP-8 (collagenase-2; **Fig. S6E**), cathepsin S (CatS), CatL, and CatK (**Fig. S6F**), were not different between *Tem1^wt/wt^* and *Tem1^lacZ/lacZ^* mice, consistent with the *in situ* zymography results (**Fig. S6G**). Thus, proteolytic activity during AAA formation was not affected by TEM1 loss. In addition, the infiltration of macrophages positive for MOMA-2, a marker of macrophages/monocytes (**Fig. 5H**), and levels of proinflammatory cytokines (**Fig. 5I**), including TNF-α, IL-6, and MCP-1, were not different between *Tem1^wt/wt^* and *Tem1^lacZ/lacZ^* mice. Staining using dihydroethidium, an oxidant-sensitive dye, showed that oxidative stress indicated by the fluorescent signal in *Tem1^wt/wt^* mice was not different from that in *Tem1^lacZ/lacZ^* mice (**Fig. S6H**). These findings revealed that matrix-degrading proteinases, inflammatory responses, or oxidative stress were not different in *Tem1*-deficient mice and wild-type controls.

In summary, *Tem1* deficiency may lead to a more severe phenotype of AAA by reducing collagen production rather than enhancing elastinolysis and vascular inflammation.

### rTEM1 promotes phenotypic change and collagen gene expression in VSMCs and fibroblasts *in vitro*

**Figure 6:**
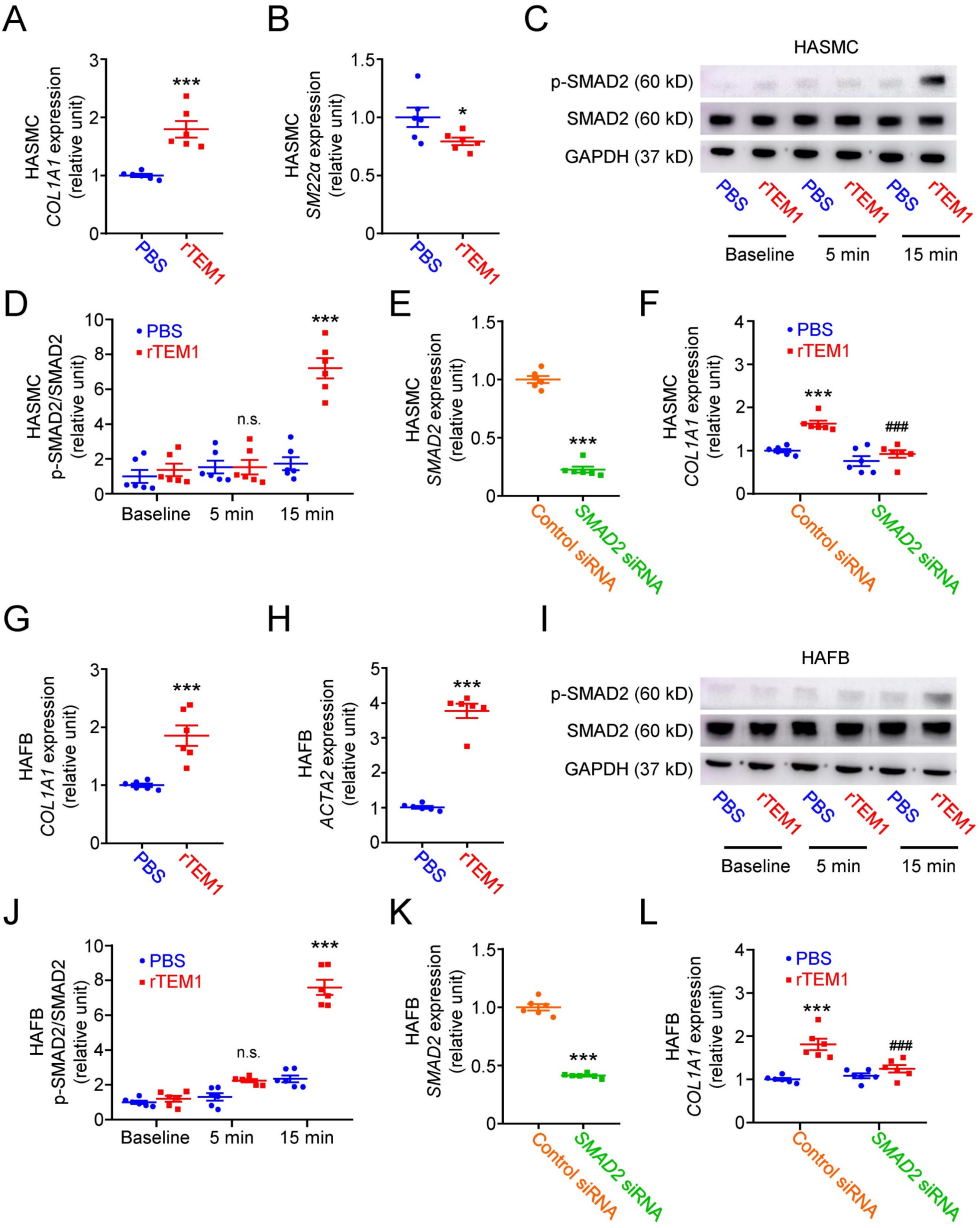
** rTEM1 enhances phenotypic change and collagen synthesis in HASMCs and HAFBs.** HASMCs (A-F) and HAFBs (G-L) were treated with PBS or 100 nM rTEM1. **(A)** *COL1A1* gene expression levels at 1 day (n=6). ***p=0.0002 vs. PBS. **(B)** *SM22α* gene expression levels at 1 day (n=6). *p=0.0443 vs. PBS. **(C)** Representative western blot analysis of p-SMAD2 and SMAD2 levels at indicated times. **(D)** Quantification of p-SMAD2/SMAD2 levels (n=6). p>0.9999 vs. 5 min, PBS. ***p<0.0001 vs.15 min, PBS. **(E)** *SMAD2* gene expression levels after transfection with control or *SMAD2* siRNA (n=6). ***p<0.0001 vs. control siRNA. **(F)** *COL1A1* gene expression levels at 1 day (n=6). ***p=0.0002 vs. control siRNA, PBS. ^###^p<0.0001 vs. control siRNA, rTEM1. **(G)** *COL1A1* gene expression levels at 1 day (n=6). ***p=0.0008 vs. PBS. **(H)** *ACTA2* gene expression levels at 1 day (n=6). ***p<0.0001 vs. PBS. **(I)** Representative western blot analysis of p-SMAD2 and SMAD2 levels at indicated times. **(J)** Quantification of p-SMAD2/SMAD2 levels (n=6). p=0.6557 vs. 5 min, PBS. ***p<0.0001 vs. 15 min, PBS. **(K)** *SMAD2* gene expression levels after transfection with control or *SMAD2* siRNA (n=6). ***p<0.0001 vs. control siRNA. **(L)** *COL1A1* gene expression levels at 1 day (n=6). ***p<0.0001 vs. control siRNA, PBS. ^###^p=0.0009 vs. control siRNA, rTEM1. (Data are represented as mean values ± SEM.)

Soluble rTEM1 was prepared to explore the function of TEM1 in AAA. Treatment with rTEM1 only (without TGF-β1 stimulation) increased *COL1A1* (**Fig. 6A**) and reduced *SM22α* gene expression (**Fig. 6B**) in HASMCs, suggesting that rTEM1 promoted collagen synthesis and VSMC switch to the synthetic phenotype. Analysis of intracellular signaling revealed that rTEM1 (without TGF-β1) did not induce the phosphorylation of SMAD2 in HASMCs at 5 minutes but at 15 minutes (**Fig. 6C & 6D**). Silencing of the *SMAD2* gene was achieved by siRNA transfection in HASMCs (**Fig. 6E**). rTEM1 increased the expression level of *COL1A1* in control siRNA-transfected HASMCs, and the increase was attenuated in *SMAD2* siRNA-transfected HASMCs, suggesting that rTEM1-induced *COL1A1* expression was mediated by SMAD2 signaling (**Fig. 6F**). Activation of TGF-β receptor (TGFBR) signaling can trigger SMAD2 phosphorylation 34. Thus, a small molecule TGFBR antagonist, SB-431542, was used to ascertain whether the effects of rTEM1 on HASMCs are mediated through TGFBR. Treatment with SB-431542 suppressed rTEM1-induced SMAD2 phosphorylation (**Fig. S7A & S7B**) and *COL1A1* expression (**Fig. S7C**) in HASMCs.

In HAFBs, rTEM1 treatment only (without TGF-β1 stimulation) induced *COL1A1* (**Fig. 6G**) and *ACTA2* gene expression (**Fig. 6H**), suggesting that rTEM1 enhanced collagen synthesis and fibroblast differentiation into myofibroblasts. Consistent with the analysis of HASMCs, rTEM1 (without TGF-β1) did not induce SMAD2 phosphorylation at 5 minutes but at 15 minutes (**Fig. 6I & 6J**). The *SMAD2* gene in HAFBs was silenced by siRNA transfection (**Fig. 6K**). rTEM1 increased the expression level of *COL1A1* in control siRNA-transfected HAFBs, and this increase was inhibited in *SMAD2* siRNA-transfected HAFBs (**Fig. 6L**). Thus, rTEM1-induced *COL1A1* expression in HAFBs was mediated by SMAD2 signaling. Whether rTEM1-induced SMAD2 phosphorylation and *COL1A1* expression in HAFBs are mediated through TGFBR was examined. Treatment with SB-431542 blocked rTEM1-induced SMAD2 phosphorylation (**Fig. S7D & S7E**) and *COL1A1* expression (**Fig. S7F**) in HAFBs. In summary, these findings suggested that rTEM1 promoted VSMC and fibroblast phenotypic change and collagen gene expression through SMAD2 signaling, and these actions were likely mediated by TGFBR.

### Treatment with rTEM1 promotes collagen synthesis and suppresses aneurysm formation

**Figure 7:**
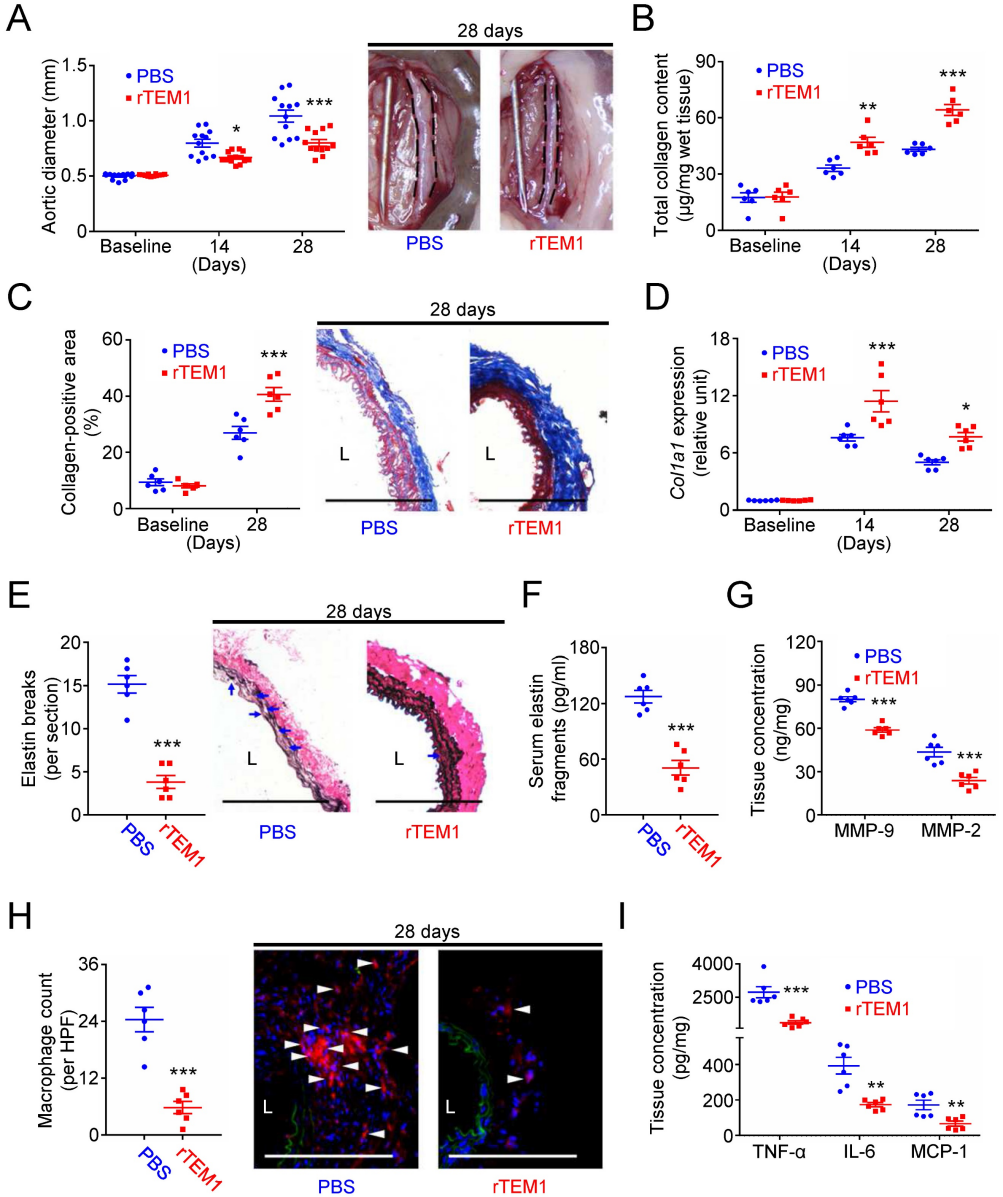
** Treatment with rTEM1 promotes collagen production in the aortic wall and attenuates CaCl_2_-induced AAA formation. (A)** Aortic diameter change (n=12 per time point) and representative microscopic photos of the infrarenal aorta. *p=0.0480, ***p<0.0001 vs. PBS.** (B)** Total collagen content (n=6 per time point). **p=0.0035, ***p<0.0001 vs. PBS. **(C)** Collagen-positive area (n=6 per time point) and representative microscopic images of Masson's trichrome staining. ***p=0.0002 vs. PBS. **(D)** *Col1a1* gene expression (n=6 per time point). ***p=0.0002, *p=0.0152 vs. PBS. **(E)** Elastin break counts (n=6) and representative microscopic images of VVG staining. Blue arrows indicate disrupted elastic lamella. ***p<0.0001 vs. PBS. **(F)** Circulating elastin fragments at 28 days (n=6). ***p<0.0001 vs. PBS. **(G)** Tissue concentration of MMP-9 and MMP-2 at 28 days (n=6). For MMP-9 and MMP-2, ***p<0.0001 and ***p=0.0006 vs. PBS, respectively. **(H)** MOMA-2-positive macrophage numbers and representative microscopic images of MOMA-2 staining (with DAPI). White arrowheads indicate MOMA-2-positive, DAPI-stained macrophages. ***p<0.0001 vs. PBS. **(I)** Tissue concentration of TNF-α, IL-6, and MCP-1 at 28 days (n=6). For TNF-α, IL-6, and MCP-1, ***p=0.0004, **p=0.0010, and **p=0.0057 vs. PBS, respectively. (L indicates lumen. All scale bars represent 100 μm. Data are represented as mean values ± SEM).

Finally, the effects of rTEM1 treatment were evaluated in two AAA models. In the CaCl_2_-induced AAA model, aortic dilatation was observed in rTEM1-treated and PBS-treated mice (**Fig. 7A**). However, the aortic diameter was smaller in rTEM1-treated mice than in PBS-treated mice at 14 days (0.67 ± 0.02 vs. 0.80 ± 0.03 mm; p=0.048, 2-way ANOVA followed by Bonferroni correction) and 28 days (0.80 ± 0.03 mm vs. 1.04 ± 0.06 mm; p<0.0001), respectively. The systolic blood pressure measured at 28 days was not different in PBS-treated and rTEM1-treated mice (**Fig. S8A**). Total collagen content (**Fig. 7B**) was increased in rTEM1-treated mice compared with PBS-treated mice at 14 and 28 days, consistent with the results of Masson's trichrome staining at 28 days (**Fig. 7C**). Picrosirius red staining revealed that the collagen fibers in rTEM1-treated mice were densely packed compared with those in PBS-treated mice (**Fig. S8B**). Also, *Col1a1* expression was higher in rTEM1-treated mice than in PBS-treated mice at 14 and 28 days (**Fig. 7D**), suggesting collagen synthesis enhanced by rTEM1 treatment. AFM indentation results showed that aortic stiffness was enhanced in rTEM1-treated mice compared with PBS-treated mice (**Fig. S8C**). The non-lesion site of the abdominal aorta was examined to realize possible off-target effects of rTEM1. Analysis of the suprarenal abdominal aorta (i.e., the non-lesion site) revealed that the collagen content (**Fig. S8D**) and collagen-positive area (**Fig. S8E**) were not increased in rTEM1-treated mice compared with PBS-treated mice, suggesting that rTEM1 treatment did not enhance collagen synthesis at the non-lesion site. Whether rTEM1 treatment affected ECM degradation and inflammatory responses that underpin the development of AAA was examined. Compared with those in PBS-treated mice, elastin breaks demonstrated by VVG staining (**Fig. 7E**) and circulating elastin fragments (**Fig. 7F**) were reduced in rTEM1-treated mice at 28 days. Aortic levels of MMP-9 and MMP-2 were suppressed by rTEM1 treatment (**Fig. 7G**). Also, macrophage infiltration (**Fig. 7H**) and levels of proinflammatory TNF-α, IL-6, and MCP-1 (**Fig. 7I**) were attenuated by rTEM1 treatment. Therefore, treatment with rTEM1 inhibited CaCl_2_-induced AAA, increased collagen content, and suppressed proteolysis and inflammatory responses.

**Figure 8:**
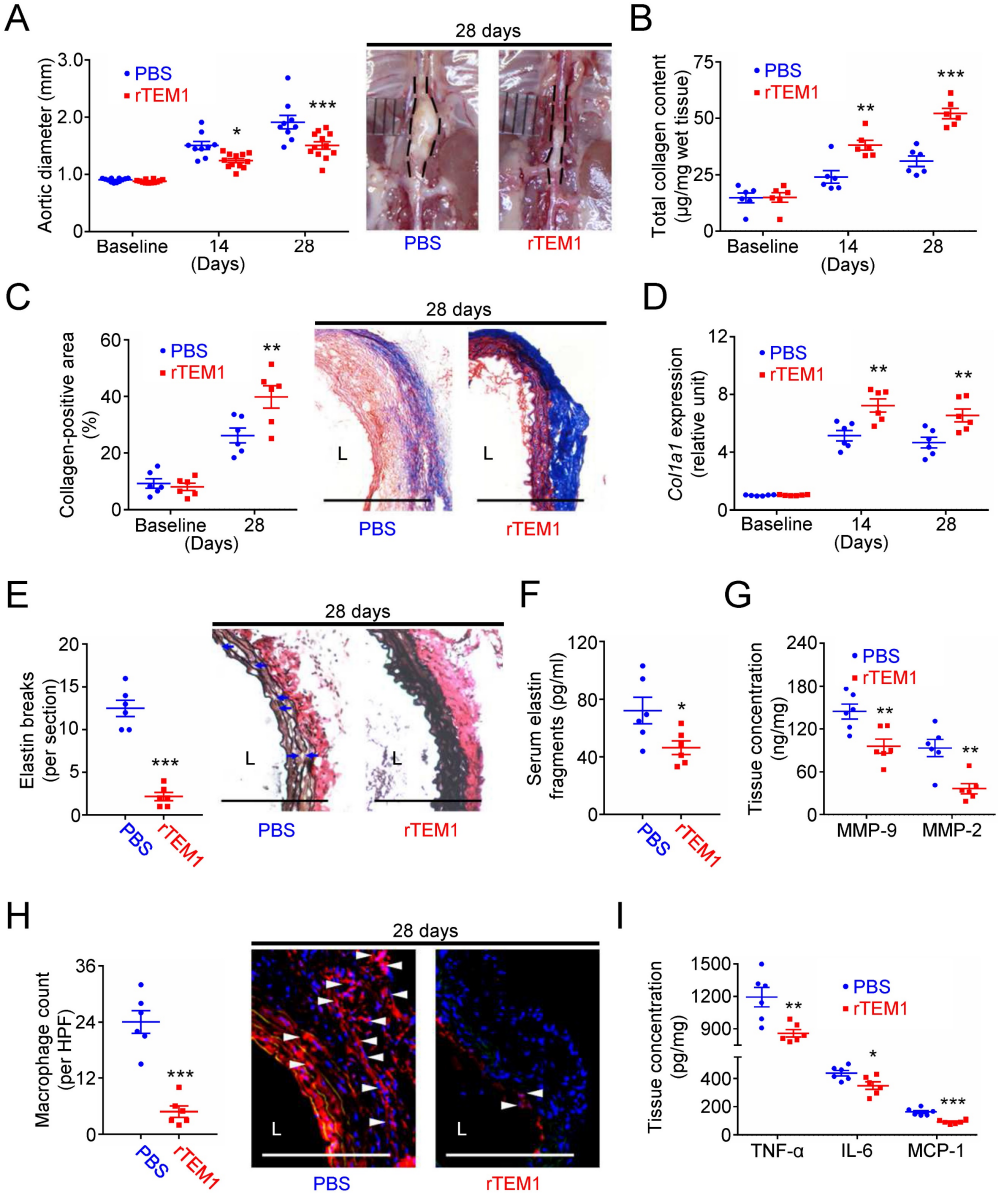
** Treatment with rTEM1 increases aortic collagen production and suppresses AngII-infused AAA. (A)** Aortic diameter change (n=9-12 per time point) and representative microscopic photos of the suprarenal aorta. *p=0.0291 and ***p=0.0001 vs. PBS. **(B)** Total collagen content (n=6 per time point). **p=0.0028, ***p<0.0001 vs. PBS. **(C)** Collagen-positive area (n=6 per time point) and representative microscopic images of Masson's trichrome staining. **p=0.0086 vs. PBS. **(D)** *Col1a1* gene expression (n=6 per time point). **p=0.0019 and **p=0.0062 vs. PBS. **(E)** Elastin break counts (n=6) and representative microscopic images of VVG staining. Blue arrows indicate disrupted elastic lamella. ***p<0.0001 vs. PBS. **(F)** Circulating elastin fragments at 28 days (n=6). *p<0.0313 vs. PBS. **(G)** Tissue concentration of MMP-9 and MMP-2 at 28 days (n=6). For MMP-9 and MMP-2, **p=0.0068 and **p=0.0023 vs. PBS, respectively. **(H)** MOMA-2-positive macrophage numbers and representative microscopic images of MOMA-2 staining (with DAPI). White arrowheads indicate MOMA-2-positive, DAPI-stained macrophages. ***p<0.0001 vs. PBS. **(I)** Tissue concentration of TNF-α, IL-6, and MCP-1 at 28 days (n=6). For TNF-α, IL-6, and MCP-1, **p=0.0060, *p=0.0245, and ***p=0.0001 vs. PBS, respectively. (L indicates lumen. Blue arrows indicate disrupted elastic lamella. All scale bars represent 100 μm. Data are represented as mean values ± SEM).

Results were further verified using the angiotensin II (AngII)-infused AAA model. The aortic diameter was increased in rTEM1-treated and PBS-treated mice (**Fig. 8A**). During the 28 days, deaths due to aortic rupture occurred less frequently in rTEM1-treated mice (0 in 24 mice) than in PBS-treated mice (6 in 24 mice; p=0.02). In addition, the aortic diameter was reduced in rTEM1-treated mice compared with PBS-treated mice on day 14 (1.24 ± 0.04 [n=12] vs. 1.50 ± 0.07 mm [n=9]; p=0.0291, 2-way ANOVA followed by Bonferroni correction) and day 28 (1.51 ± 0.06 mm [n=12] vs. 1.91 ± 0.12 mm [n=9]; p=0.0001). The systolic blood pressure measured at 28 days was not different in PBS-treated and rTEM1-treated mice (**Fig. S9A**).

Analysis of aortic samples showed that collagen content was higher in rTEM1-treated mice than in PBS-treated mice at 14 and 28 days (**Fig. 8B**), consistent with the collagen-positive area at 28 days shown by Masson's trichrome staining (**Fig. 8C**). Picrosirius red staining revealed that the collagen fibers in rTEM1-treated mice were densely packed and well organized compared with those in PBS-treated mice (**Fig. S9B**). Additionally, *Col1a1* expression was higher in rTEM1-treated mice than in PBS-treated mice at 14 and 28 days (**Fig. 8D**).

AFM indentation revealed that aortic samples were stiffer in rTEM1-treated mice than in PBS-treated mice (**Fig. S9C**). Analysis of the infrarenal abdominal aorta (i.e., the non-lesion site) showed that the collagen content (**Fig. S9D**) and collagen-positive area (**Fig. S9E**) were not increased under rTEM1 treatment. The effects of rTEM1 treatment on proteolytic degradation of ECM and inflammatory responses were also evaluated. The elastin break counts (**Fig. 8E**) were lower in rTEM1-treated mice than in PBS-treated mice at 28 days, consistent with the circulating levels of elastin fragments (**Fig. 8F**) and aortic levels of MMP-9 and MMP-2 (**Fig. 8G**). Macrophage infiltration (**Fig. 8H**) and aortic levels of TNF-α, IL-6, and MCP-1 (**Fig. 8I**) were attenuated in rTEM1-treated mice compared with PBS-treated mice. In line with the observations of the CaCl_2_-induced model, treatment with rTEM1 attenuated AngII-infused AAA formation, with enhanced collagen synthesis and reduced proteolytic activity and inflammatory responses.

## Discussion

The development of AAA involves complicated pathological mechanisms. Substantial interest exists in better understanding the pathogenesis to identify novel therapeutic approaches that inhibit aneurysm development. Gaining insights into critical pathogenic mechanisms still poses a principal challenge in establishing potential pharmacological approaches for AAA. In this study, we have investigated the role of TEM1 in AAA. TEM1 expression was upregulated in human and mouse AAA and was derived mainly from VSMCs and fibroblasts. Bioinformatics analysis identified the link between TEM1 and collagen and TEM1-related candidate cell populations. *In vitro* and *in vivo* studies demonstrated that TEM1 loss reduced collagen synthesis in VSMCs and fibroblasts and intensified CaCl_2_-induced aortic dilatation. In contrast, administration of rTEM1 increased collagen synthesis and suppressed AAA in two different models. Structural collagen in the media and adventitia provides the mechanical strength of the aortic wall 6, 7. Our study suggests that TEM1 regulates phenotypic change and collagen synthesis in VSMCs and fibroblasts, stabilizing structural integrity against aortic expansion. These observations may provide new insights leading to innovative therapeutic strategies for AAA.

TEM1 is not expressed or found at very low levels in adult tissues 14. Characterization of human samples showed that TEM1 was hardly found in normal aortas. In AAA, TEM1 appeared in medial VSMCs and adventitial fibroblasts. Dissection of the animal model revealed elevated TEM1 expression and collagen content during AAA formation. Integrating bioinformatics tools into translational research may facilitate more efficient drug discovery 35, 36. Bioinformatics has been applied for functional understanding of the human genome, leading to the enhanced discovery of target signaling pathways. High-throughput information obtained from previously published data indicated TEM1-related disorders and the link between TGF-β signaling and VSMCs and fibroblasts, both of which are resident stromal cells responsible for collagen synthesis 37, 38.

TEM1 is identified as a target or possesses therapeutic potential in fibrosis-related processes such as wound healing and organ fibrosis 14, 24-27, 39. *TEM1* deletion has been shown to reduce collagen deposition in these physiological and pathological mechanisms. While profibrotic responses that lead to organ dysfunction are often considered pathological, pharmacological intervention and genetic manipulation to enhance collagen production have displayed encouraging results for controlling AAA formation 12, 40-42. In this study, *TEM1* knockdown inhibited VSMC and fibroblast phenotypic transformation and collagen synthesis stimulated by TGF-β/SMAD2 signaling. *Tem1* deficiency reduced collagen production, and loss of mechanical strength provided by collagen exacerbated aortic dilatation after AAA induction in mice. Of note, collagen is highly resistant to proteolytic degradation, and only a few collagenases in the MMP and cysteine cathepsin families are mammalian proteinases capable of cleaving the triple-helical structure of collagen 8. As no difference was observed in collagenase levels between *Tem1*-deficient mice and wild-type controls, the reduced collagen deposition observed in *Tem1*-deficient mice may be attributed to suppressed collagen synthesis instead of increased collagen degradation. It is plausible to postulate that impairment of collagen synthesis as an endogenous repair mechanism aggravated aortic dilatation in *Tem1*-deficient mice. This notion is further consolidated because TEM1 loss did not interfere with elastin fragmentation, inflammatory responses, and oxidative stress, all of which are typical characteristics observed in AAA 3, 4, 8.

Restoration or maintenance of ECM integrity has been proposed as an effective therapeutic perspective to suppress aortic dilatation. Such a strategy can be achieved by several methods 12, 31, 43, including administration of locked nucleic acid anti-microRNA-29b, intramural delivery of TGF-β1 hydrogel, or periadventitial administration of nontoxic stabilizing agents such as pentagalloyl glucose. This approach stands apart from general concepts in developing potential pharmacotherapies of AAA, which often focus on targeting inflammatory processes, oxidative stress, or proteolytic degradation of ECM 5. In the present study, administration of rTEM1 promoted VSMC and fibroblast phenotypic transformation, leading to increased collagen synthesis *in vitro* and *in vivo*. Intriguingly, while TEM1 loss did not disturb elastin fragmentation and inflammatory responses—the primary characteristics of AAA—we observed a notable reduction in these features in mice treated with rTEM1. Probably because of the proper mechanical environment and structural integrity preserved by adequate collagen materials, the release of biologically active peptides (i.e., elastin fragments) that may exert monocyte chemotactic activity 44, 45 was diminished, impeding the perpetuation of proteolytic degradation and inflammation. Matrix-degrading proteinases were also reduced, given that matrix stiffness and collagen content can regulate lesion MMP production 46. Consequently, collagen synthesis amplified by rTEM1 treatment provides a mechanical reinforcement to protect matrix architecture and thus may hold promise as a strategy to treat AAA.

Our study has some limitations and potential future works. First, medial VSMCs and adventitial fibroblasts are resident cells essential for maintaining the structure of the aortic wall, and both undergo phenotypic switch and increase ECM synthesis in AAA 47, 48. The present study demonstrated that TEM1 is derived from both VSMC and fibroblasts during AAA formation, and global TEM1 deletion can affect aortic remodeling processes. Given that TEM1 regulates phenotypic change and collagen gene expression in VSMCs and fibroblasts *in vitro*, the differential impact of TEM1 in VSMCs and fibroblasts on collagen synthesis *in vivo* remains to be determined. Transgenic mice with tissue-specific loss of TEM1 may be required to answer this question. Second, along with previous studies 12, 40-42, our data revealed that enhancing collagen synthesis can be a promising approach for treating AAA. In some clinical experiences, fibrotic scars from previous cardiac surgery can exert protective effects to contain lethal aortic rupture 49, 50. In the present study, aortic stiffness of the aneurysm site was enhanced by rTEM1-induced profibrotic responses. Systolic blood pressure was not altered, possibly because rTEM1-enhanced collagen synthesis and aortic stiffness were observed only at a short segment of the abdominal aorta (i.e., the aneurysm site). Notably, previous studies have shown that increased collagen synthesis in the aortic wall may enhance arterial stiffness, predisposing individuals to hypertension and atherosclerotic cardiovascular diseases 7, 51. Balancing potential positive and negative side effects while using this strategy is thus a critical issue. Further studies on the optimal control of TEM1-mediated collagen synthesis in the aortic wall are warranted to maximize the benefits and minimize the harm. Third, consistent with the general understanding that TEM1 plays a role in remodeling during tissue repair 14, our study showed that administration of rTEM1 can be a novel approach to treat AAA. However, triggering collagen synthesis in other organs, such as the liver or kidney, is unfortunately associated with dysfunction of these organs 24-26, implying that systemic rTEM1 administration per se will not be ideal for translational approaches in humans. In this context, although rTEM1 treatment did not increase collagen content at the non-lesion site of the abdominal aorta, local delivery of rTEM1 to trigger collagen synthesis in the aortic wall may be more promising as a therapeutic approach to treat a small AAA and thus needs further studies. Finally, although aortic aneurysm and dissection have distinct pathological mechanisms and clinical presentations, they share similar pathogenesis, such as vascular inflammation and impaired structural integrity and mechanical properties 52. In the present study, the prediction of human gene-phenotype associations (**Fig. 2A**) revealed that *TEM1* gene was linked to aortic dissection and aneurysm, and the risk is slightly higher in aortic dissection (Z-score: 5.251766) than in aortic aneurysm (Z-score: 4.262546). Given that TEM1 in resident stromal cells regulates collagen synthesis during AAA formation, TEM1 is also expected to play a role during the development of aortic dissection. Interestingly, Maegdefessel *et al.* demonstrated that increased collagen synthesis by microRNA-29b inhibition reduces aneurysm progression and lethal rupture and dissection, suggesting that profibrotic responses to maintain vascular integrity may help hinder both aortic aneurysm and dissection 12. Thus, it is tempting to explore the role of TEM1 in aortic dissection in the future.

## Conclusions

In conclusion, we have demonstrated the significance of TEM1 in AAA. Our study suggests that TEM1 loss leads to impairment of collagen synthesis and exacerbation of aortic expansion, whereas administration with rTEM1 may be potentially beneficial in protecting the aorta from expansion and ultimate rupture. During AAA formation, TEM1 expression in resident stromal cells emerges as a compensatory protective mechanism that stabilizes structural integrity of the aortic wall. This function is quite distinct from the well-known pathologic role of TEM1 in cancer and organ fibrosis. Although the safety profile of rTEM1 needs to be further evaluated, our findings suggest that matrix reloading promoted by rTEM1 treatment might be a potential therapeutic approach for AAA.

## Methods

### Human specimens

Human AAA specimens were retrospectively obtained from 8 patients who underwent conventional open surgical repair. Paraffin-embedded tissue sections were retrieved from the Human Tissue Bank, Clinical Medicine Research Center in National Cheng Kung University Hospital. Informed consent was waived because the AAA specimens were de-identified. Eight normal human aorta specimens obtained from autopsy (US Biomax, Rockville, MD; OriGene Technologies, Rockville, MD; Pantomics, Richmond, CA) were used as controls for comparison. None of these individuals were known to have AAA or underlying connective tissue diseases. Immunohistochemical analysis with TEM1 antibody (18160-1-AP; Proteintech, Rosemont, IL) was performed using the Bond-Max automated immunohistochemistry stainer (Leica Biosystems, Melbourne, Australia). Counterstaining was performed with hematoxylin. The area of TEM1 expression was determined using HistoQuest software. Immunostaining for FSP-1 (sc-376987; Santa Cruz Biotechnology, Santa Cruz, CA), a marker of fibroblasts, and α-SMA (A2547; Sigma-Aldrich, St. Louis, MO) was performed to examine the colocalization of TEM1 with fibroblasts or VSMCs in AAA specimens. The human study was approved by the Institutional Review Board of National Cheng Kung University Hospital (approval number: A-ER-106-235).

### Animals

C57BL/6 mice and ApoE^-/-^ mice (stock number: 002052) were purchased from the National Laboratory Animal Center in Taiwan and Jackson Laboratory (Bar Harbor, ME), respectively. *Tem1*-deficient *Tem1^lacZ/lacZ^* mice in which the exon of *Tem1* gene was replaced with *lacZ* reporter gene were obtained from Dr. Shu-Wha Lin at National Taiwan University Hospital and genotyped as previously described 53. *Tem1^lacZ/lacZ^* mice were viable and fertile without any known congenital anomalies during development or after birth 54.

### Cell culture

Primary HASMCs and HAFBs were purchased from Lonza Group (Basel, Switzerland). The culture medium and conditions were determined according to the commercial protocol. TGF-β1 (R&D Systems, Minneapolis, MN) was added at indicated doses, and the siRNAs targeting *TEM1* and *SMAD2* (Dharmacon, Lafayette, CO) were transfected to interfere with gene expression. SB-431542 (Cayman Chemical, Ann Arbor, MI) was added at indicated doses to inhibit TGFBR activation.

### Expression of soluble rTEM1 ectodomain protein

As previously described 55, the pPICZaA and pCR3-EK vectors (Invitrogen, Carlsbad, CA) were used to express and secrete recombinant TEM1 functional domains, which included a 6-peat His tag and c-Myc epitope for purification and detection purposes. Human embryonic kidney 293 cells serve as the mammalian protein expression system. The purified rTEM1 proteins were analyzed by Coomassie blue staining and Western blotting following gel electrophoresis.

### AAA models and rTEM1 treatment

As described previously with minor modifications 56, 57, the AAA model was induced by CaCl_2_ in C57BL/6, *Tem1^lacZ/lacZ^*, and *Tem1^wt/wt^* mice at 8 to 10 weeks of age. After laparotomy, the abdominal aorta was exposed, and a small piece of cotton gauze (0.3 × 0.3 cm^2^) soaked in 0.5 M CaCl_2_ solution was applied periaortically for 15 minutes. At indicated time points, the mice were sacrificed, and the infrarenal aortic diameter was measured using a digital caliper. The whole segment of the abdominal aorta, including the infrarenal aorta (i.e., the aneurysm site) and suprarenal aorta (i.e., the non-lesion site), was harvested for further analysis. To establish the AngII-infused model, 6-month-old male ApoE^-/-^ mice were subcutaneously infused with AngII (1000 ng/kg/min; Sigma-Aldrich) via osmotic pumps (Alzet 2004; Durect, Cupertino, CA) 56, 57. After pump implantation, the mice were fed a Western diet (0.15% cholesterol and 21% milk fat, 57BD; TestDiet, Richmond, IN). At indicated time points, the mice were sacrificed, and the suprarenal aortic diameter was measured using a digital caliper. The whole segment of the abdominal aorta, including the suprarenal aorta (i.e., the aneurysm site) and infrarenal aorta (i.e., the non-lesion site), was harvested for further analysis.

The effect of *Tem1* deficiency was evaluated in the CaCl_2_-induced AAA model using *Tem1^lacZ/lacZ^* and *Tem1^wt/wt^* mice. The potential therapeutic effect of rTEM1 was evaluated in CaCl_2_-induced and AngII-infused AAA models using C57BL/6 and *ApoE*^-/-^ mice, respectively. Mice received treatment of rTEM1 (intraperitoneal injection with rTEM1 [360 μg/kg] in 0.2 mL PBS every three days from one day after AAA induction) or an equal volume of PBS only. The maximal diameter of aortic dilatation in each mouse was blindly determined by 2 independent observers 3 times, and the data were then averaged. The animal studies were approved by the Institutional Animal Care and Use Committee of National Cheng Kung University (approval number: 107028) and conformed to the *Guide for the Care and Use of Laboratory Animals* published by the National Institutes of Health (NIH Publication #85-23, revised 1996).

### Measurement of systolic blood pressure

The blood pressure was recorded using the non-invasive tail-cuff device (BP-2000 System; Visitech Systems, Apex, NC). Mice were adapted to the device for 3 days. Systolic blood pressure was measured at 28 days before sacrifice. Each mouse received six measurements, and the average value was obtained for comparison.

### Measurement of aortic stiffness by AFM

Aortic stiffness was measured as previously described 58. Transverse cryosectioned (20 μm thick) aortic tissue was prepared to measure aortic stiffness using polystyrene bead (25 μm in diameter)-modified CSC12-F tipless AFM cantilevers (MikroMasch, Wetzlar, Germany). The spring constants of all cantilevers were 0.08 N/m and calibrated via the thermal noise method in liquid prior to each measurement. The indenting force was set at 1 to 3 nN. The approaching and retracting cantilever rates were set at 1 μm/second. Force-distance curves were collected and calculated using JPK package software, which was based on the Hertz model. At least 30 measurements of the Young's Modulus were obtained from each aorta, and the average value was used for comparison.

### Western blotting

Cells were harvested in a lysis buffer. The total protein (20 μg) was separated by 10% sodium dodecyl sulfate‒polyacrylamide gel electrophoresis and transferred to a polyvinylidene difluoride membrane. The membrane was blocked using 5% non-fat dry milk for 1 hour at room temperature. The membrane was hybridized with primary antibodies against TEM1 (18160-1-AP; Proteintech), SM22α (GTX113561; GeneTex), SMAD2 (sc-133098; Santa Cruz Biotechnology) and p-SMAD2 (8828S; Cell Signaling, Danvers, MA) overnight at 4°C and washed with PBST before being incubated with horseradish peroxidase-conjugated secondary antibody (AP124P and AP307P; Invitrogen) for 2 hours at room temperature. An enhanced chemiluminescence reagent (Millipore, Temecula, MA) was used to detect the signal captured by the ImageQuant LAS 4000 mini imager (GE Healthcare, Chicago, IL). Quantification of band intensity was performed using ImageJ software.

### Enzyme-linked immunosorbent assay (ELISA)

Serum was isolated after clots were removed from blood samples and was used to measure circulating elastin fragments. Complete samples from CaCl_2_-induced AAAs and the upper 1/3 of samples from AngII-infused AAAs were used for biochemical analysis of the aortic samples. The remaining 2/3 of the samples from AngII-infused AAAs were used for histological analysis because the maximal expansion was typically located at the middle part of the aortic samples. After the samples were disrupted, aortic homogenates were obtained using a sonicator (Sonicator 3000; Misonix, Farmingdale, NY). Radioimmunoprecipitation assay buffer (RIPA Buffer; Abcam, Cambridge, MA) was used to perform protein extraction. ELISA was used to measure the level of circulating elastin fragments (Taiclone, Taipei, Taiwan) in the serum and tissue levels of TEM1 (Cusabio, Houston, TX), MMP-9, MMP-2 (Abnova, Taipei, Taiwan), CatS, CatL, CatK (Cloud-Clone, Katy, TX), TNF-α, IL-6, and MCP-1 (R&D Systems) in mouse aortic homogenates based on the manufacturers' instructions. The amount of each protein in each sample was measured by spectrophotometric optical density (450 nm) using an automated microplate reader (SpectraMax 340PC384; Molecular Devices, Sunnyvale, CA).

### Total collagen assay

Total collagen assay (BioVision, Milpitas, CA) was used to measure the total collagen content in the aortic specimens based on the manufacturer's instructions. The assay is based on the acid hydrolysis of samples to form hydrolysates and hydroxyproline. The amount of total collagen in each sample was measured by spectrophotometric optical density (560 nm) using an automated microplate reader (FLUOstar Omega; BMG LABTECH, Ortenberg, Germany).

### Histological analysis of mouse specimens

Histological analysis was performed as previously described 59. In brief, frozen sections were used for staining or immunostaining for TEM1 (18160-1-AP; Proteintech), α-SMA (A2547; Sigma-Aldrich), ER-TR7 (NBP1-44961; Novus Biologicals, Centennial, CO), MOMA-2 (ab33451; Abcam), VVG staining for elastin degradation (HT25A-1KT; Sigma-Aldrich), picrosirius red (ab150681; Abcam) and Masson's trichrome (ab150686; Abcam). Unfixed aortic cryosections were incubated with 40 mg/mL dye-quenched protein substrate (DQ-gelatin; Invitrogen) for 1 h at 37°C to perform *in situ* zymography. Lung sections from normal mice served as positive controls, and samples incubated with 10 mM EDTA, a MMP inhibitor, served as negative controls. For the measurement of *in situ* oxidative stress, unfixed aortic cryosections were incubated with 5 mM dihydroethidium (DHE; Invitrogen) for 30 min at 37°C. Serial sections incubated with 20 mM N-acetyl cysteine (NAC), an ROS scavenger, served as negative controls. Images were captured using a fluorescence microscope (DP73; Olympus, Tokyo, Japan). The collagen-positive area in each section was calculated using ImageJ software. The numbers of elastin breaks and MOMA-2-positive macrophages at the maximal expansion of the aortic specimens were evaluated blindly by 2 independent observers.

### Quantitative polymerase chain reaction (qPCR)

Gene expression in human cell lysates and mouse aortic specimens was evaluated using qPCR as previously described 12. SYBR qPCR assays were performed using specific primers (Thermo Fisher Scientific, Waltham, MA) for human *TEM1* (forward, 5'-AGTGTTATTGTAGCGAGGGACA-3' and reverse, 5'-CCTCTGGGAAGCTCGGTCTA-3'), *COL1A1* (forward, 5'-GAGGGCCAAGACGAAGACATC-3' and reverse, 5'-CAGATCACGTCATCGCACAAC-3'), *SM22α* (forward, 5'-AGTGCAGTCCAAAATCGAGAAG-3' and reverse, 5'-CTTGCTCAGAATCACGCCAT-3'), *ACTA2* (forward, 5'-AAAAGACAGCTACGTGGGTGA-3' and reverse, 5'-GCCATGTTCTATCGGGTACTT-3'), *SMAD2* (forward, 5'-GTCCATCTTGCCATTCACG-3' and reverse, 5'-CTCAAGCTCATCTAATCGTCCTG-3'), and mouse* Col1a1* (forward, 5'-CAGTCGATTCACCTACAGCACG-3' and reverse, 5'-GGGATGGAGGGAGTTTACACG-3'). All target genes were normalized to GAPDH as an internal control. Amplification and detection were performed on the StepOne™ Real-Time PCR System (Thermo Fisher Scientific). The relative expression was calculated using the comparative delta-delta CT (2-∆∆Ct) method.

### Filtered analysis of raw scRNA-seq data

The filtered gene expression matrices were downloaded from a published paper 29. The Seurat R package (version 3.2.2) 60 was used to analyze the scRNA-seq data. Cells with fewer than 200 genes or more than 6,000 genes detected, as well as cells with more than 10% mitochondrial genes, were excluded. Following quality control, we obtained 4,366 cells. Sequencing reads of each gene were normalized to total UMIs in each cell to acquire the normalized UMI values by the ''*NormalizeData*'' function. After merging the two scRNA-seq datasets, we used “*FindVariableFeatures*” to identify the top 2000 variable genes. The batch effect of the two datasets was eliminated by the “*IntegrateData*” function. The gene expression levels were scaled and centered using the “*ScaleData*” function. “*RunPCA*” was applied to carry out PCA dimensionality reduction. We constructed a shared nearest neighbor graph between each cell using the “*FindNeighbors*” function. We determined the cell clusters using the “*FindClusters*” function at a resolution of 0.3. Dimensionality reduction by Uniform Manifold Approximation and Projection (UMAP) was performed by the ''*RunUMAP*'' function. Differentially expressed genes of each cluster with averaged log2 fold change of 0.5 and an adjusted p value of 0.05 were determined by the default “*FindAllMarkers*” function. Differentially expressed genes in the top 10 list of each cell population were shown by the “*DoHeatmap*” function. Gene expression in each cluster or sample was determined with the “*VlnPlot*” functions. Cell types were annotated by the “*CreateSinglerObject*” function in the SingleR package. The Seurat code is provided in the **Supplementary Method 1**.

### Cell-cell communication analysis

To analyze cell-cell communication pathways between various cell populations, we used the CellChat R package (version 0.5.5) 33 to infer the intercellular communication network from scRNA-seq data. The UMI matrix in each cell population, processed by Seurat, served as the input data by the “*createCellChat*” function. The communication probability and cellular communication network were computed by the “*computeCommunProb*” function. We employed the "*filterCommunication*" function to select the ligands and receptors expressed in the top 10% rank of each cell type. The cell-cell communication in every single signaling pathway was inferred by the “*computeCommunProbPathway*” function. The major secretor and receiver in 2D space were examined by the “*netAnalysis_signalingRole_scatter*” function. The contribution of each ligand-receptor pair to a specific signaling pathway was examined by the “*netAnalysis_contribution*” function. The network in a specific signal pathway was shown by the “*netVisual_individual*” function. The network centrality score in a specific signal pathway was calculated using the “*netAnalysis_computeCentrality*” function and displayed by the “*netAnalysis_signalingRole_network*” function. The CellChat code is provided in the **Supplementary Method 2**.

### Statistical analysis

Binary data were compared using the *χ*2 or Fisher's exact test. Continuous data were presented as mean ± SEM. Student's *t*-test was used for 2-group comparisons. One-way analysis of variance was used for multi-group comparisons, followed by post hoc analysis (Bonferroni correction). Comparisons among different groups and time points pairwise were performed using 2-way analysis of variance followed by Bonferroni correction. GraphPad PRISM 6 (GraphPad Software, San Diego, CA) was used for these comparisons. Correlation between two variables was evaluated using Spearman rank correlation analysis. A p<0.05 was considered significant.

## Supplementary Material

Supplementary figures and methods.

## Abbreviations

AAA: Abdominal aortic aneurysm
ECM: extracellular matrix
TNF-α: tumor necrosis factor-α
IL-6: interleukin-6
MCP-1: monocyte chemoattractant protein-1
MMP: matrix metalloproteinase
TEM1: tumor endothelial marker 1
VSMC: vascular smooth muscle cell
rTEM1: recombinant TEM1
α-SMA: α-smooth muscle actin
FSP-1: fibroblast-specific protein-1
scRNA-seq: single-cell RNA sequencing
HASMC: human aortic smooth muscle cell
TGF-β1: transforming growth factor-β1
HAFB: human aortic adventitial fibroblast
VVG: Verhoeff Van Gieson
AFM: atomic force microscopy
CatS: cathepsin S
TGFBR: transforming growth factor-β-receptor
AngII: angiotensin II
ELISA: enzyme-linked immunosorbent assay
qPCR: quantitative polymerase chain reaction
