# Supplementary material for: Regulation of matrix reloading by tumor endothelial marker 1 protects against abdominal aortic aneurysm
Source: Int J Biol Sci. 2024 Jul 2;20(10):3691–709. doi: 10.7150/ijbs.93526 (PMC11302889; doi:10.7150/ijbs.93526)
Supplement: Supplementary file 1 — Supplementary figures and methods. [file ijbsv20p3691s1.pdf]

Supplementary Figures

A

Predicted biological processes of Gene Ontology (GO)	Z-score
Protein heterotrimerization (GO:0070208)	8.492755
Collagen fibril organization (GO:0030199)	7.2722
Bone trabecula formation (GO:0060346)	6.358775
Extracellular fibril organization (GO:0043206)	4.814142
Peptidyl-proline hydroxylation (GO:0019511)	3.851592
Extracellular matrix organization (GO:0030198)	3.21916
Extracellular structure organization (GO:0043062)	3.205665
Extracellular regulation of signal transduction (GO:1900115)	3.148673
Smooth muscle tissue development (GO:0048745)	3.009338
Stress fiber assembly (GO:0043149)	2.96951

B

Predicted pathways (KEGG)	Z-score
ECM-receptor interaction_Homo sapiens_hsa04512	4.934252
Focal adhesion_Homo sapiens_hsa04510	3.44785
Proteoglycans in cancer_Homo sapiens_hsa05205	2.499063
TGF-β signaling pathway_Homo sapiens_hsa04350	1.851262

Fig. S1. *TEM1* is associated with extracellular matrix organization. (A) Predicted Gene Ontology (GO) biological processes. **(B)** Kyoto Encyclopedia of Genes and Genomes (KEGG) pathways.

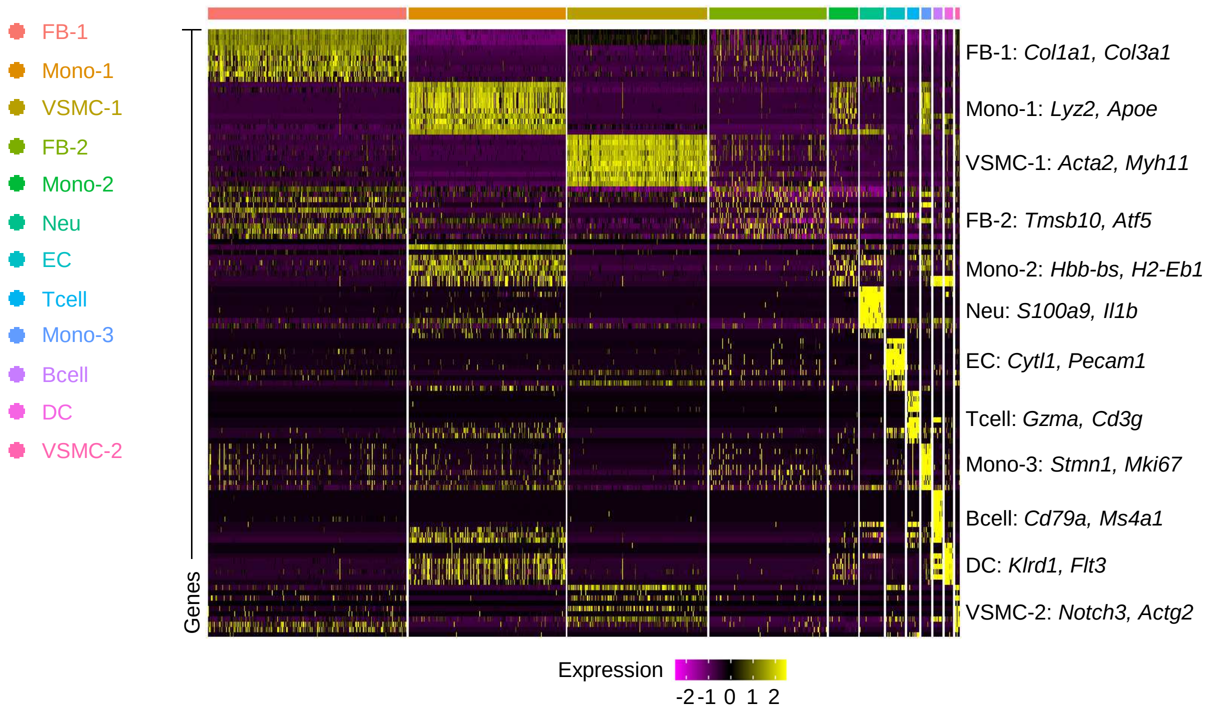

Fig. S2. Cellular heterogeneity in mouse AAA tissues is identified by scRNA-seq. The heatmap shows the top 10 differentially expressed genes in each cell type. Selected genes for each cluster are shown on the right side. (FB, fibroblast; VSMC, vascular smooth muscle cell; Mono, monocyte; Neu, neutrophil; EC, endothelial cell; Tcell, T cell; Bcell, B cell; DC, dendritic cell.**)**

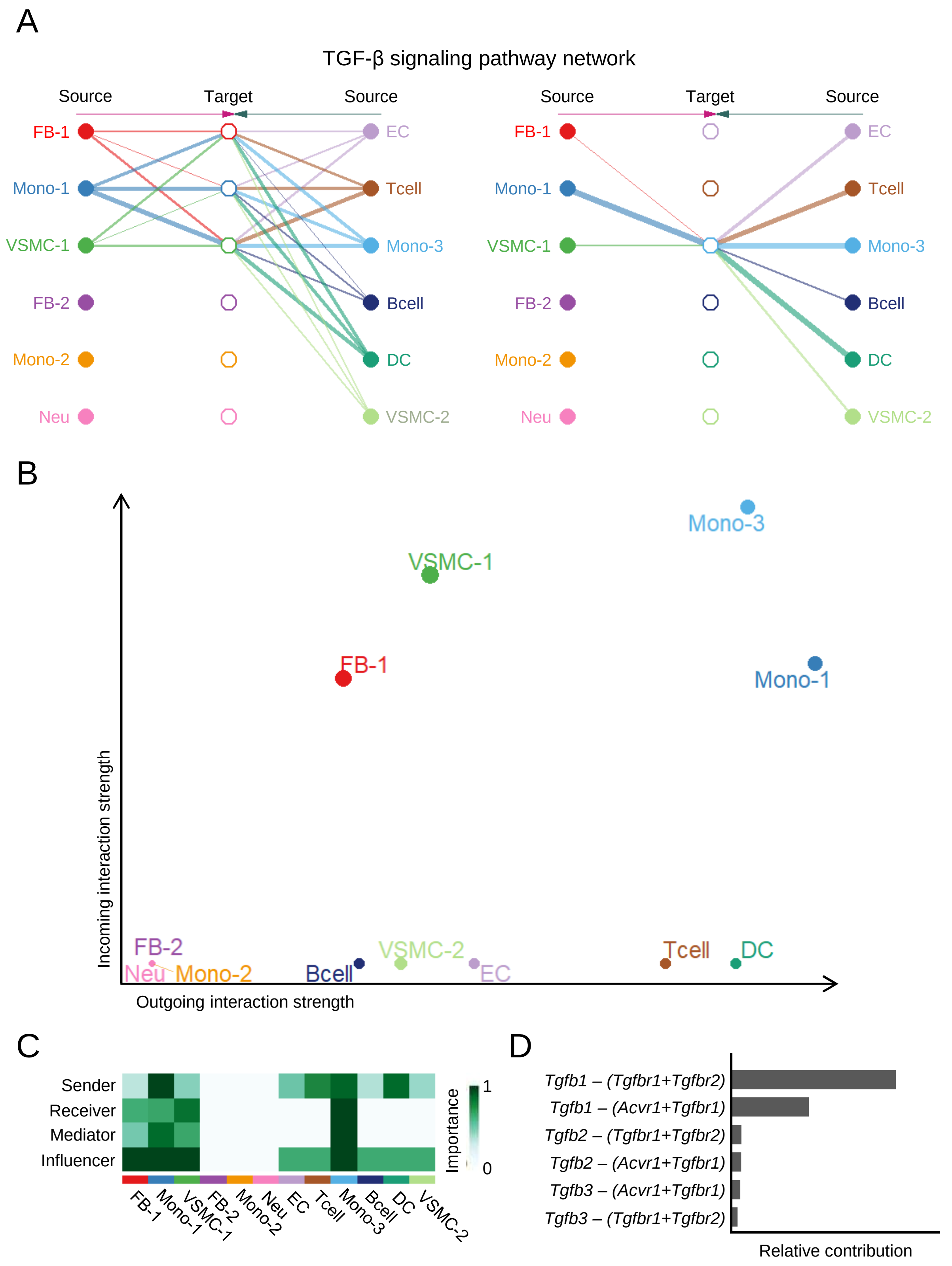

Fig. S3. VSMCs and fibroblasts are linked to TGF-β signaling. (A) Autocrine and paracrine TGF-β signal transduction pathways among all groups are visualized using a hierarchical plot. **(B)** The outgoing and incoming interaction strength displays the significant shifts in secreting and receiving signal respectively in each cluster. **(C)** Heatmap shows the relative importance of each cell group based on the computed four network centrality measures of TGFβ signaling network. **(D)** The relative contribution of each ligand-receptor pair in TGF-β signaling pathway is computed. (FB, fibroblast; VSMC, vascular smooth muscle cell; Mono, monocyte; Neu, neutrophil; EC, endothelial cell; Tcell, T cell; Bcell, B cell; DC, dendritic cell.)

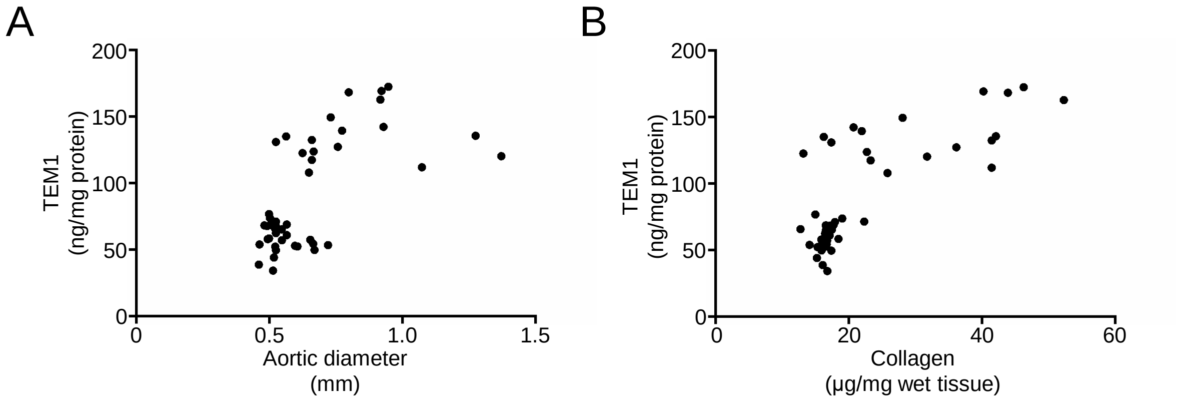

Fig. S4. Correlation analysis between aortic diameters and TEM1 and between collagen content and TEM1 during mouse AAA formation. (A) Correlation between aortic diameters and TEM1. Spearman correlation coefficient=0.5607. $p<0.0001$. **(B)** Correlation between collagen content and TEM1. Spearman correlation coefficient=0.7162. $p<0.0001$.

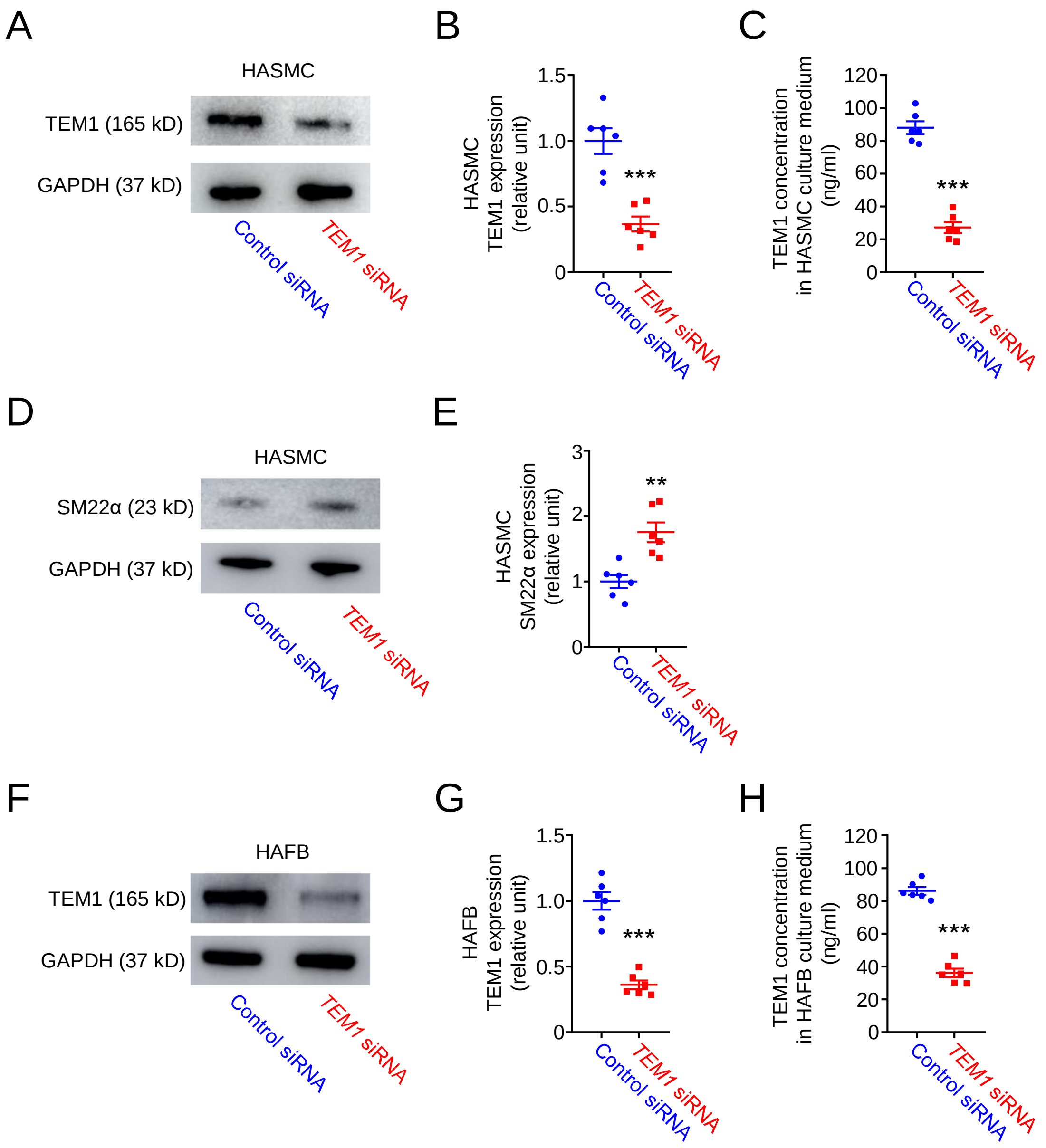

Fig. S5. *TEM1* silencing inhibits TEM1 protein expression in HASMCs and HAFBs and enhances SM22α protein expression in HASMCs. HASMCs (A-E) and HAFBs (F-J), transfected with control or *TEM1* siRNA, were treated with 10 ng/ml TGF-β1. **(A)** Representative western blot analysis of TEM1 protein levels. **(B)** Quantification of TEM1 protein levels (n=6). ***p=0.0002 vs. control siRNA. **(C)** TEM1 concentration in HASMC culture medium. ***p<0.0001 vs. control siRNA. **(D)** Representative western blot analysis of SM22α protein levels at 1 day. **(E)** Quantification of SM22α protein levels (n=6). **p=0.0020 vs. control siRNA. **(F)** Representative western blot analysis of TEM1 protein levels. **(G)** Quantification of TEM1 protein levels (n=6). ***p<0.0001 vs. control siRNA. **(H)** TEM1 concentration in HAFB culture medium. ***p<0.0001 vs. control siRNA. (Data are represented as mean values ± SEM.)

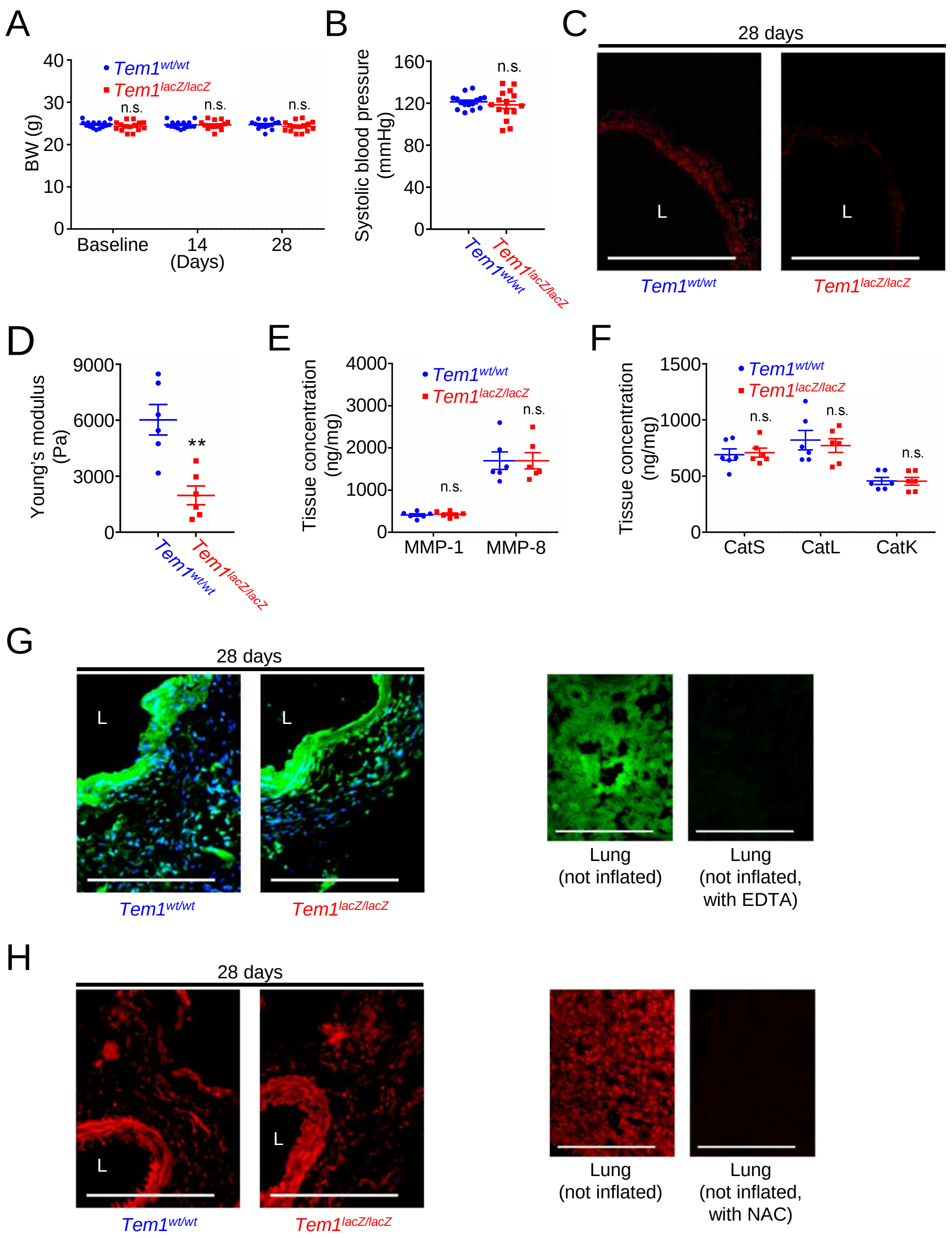

Fig. S6. TEM1 does not regulate production of proteinases and oxidative stress during aneurysm formation. (A) Body weight change (n=15 per time point). For baseline, 14 days, and 28 days, n.s. p=0.2239, n.s. p=0.8471, and n.s. p=0.2636 vs. *Tem1*$^{wt/wt}$, respectively. **(B)** Systolic blood pressure at 28 days (n=15). n.s. p=0.4665 vs. *Tem1*$^{wt/wt}$. **(C)** Representative microscopic images of picrosirius red staining. **(D)** AFM indentation results of aortic samples at 28 days (n=6). **p=0.0018 vs. *Tem1*$^{wt/wt}$. **(E)** Tissue concentration of MMP-1 and MMP-8 at 28 days (n=6). For MMP-1 and MMP-8, n.s. p=0.7094 and n.s. p=0.9995 vs. *Tem1*$^{wt/wt}$, respectively. **(F)** Tissue concentration of CatS, CatL, and CatK at 28 days (n=6). For CatS, CatL, and CatK, n.s. p=0.7914, n.s. p=0.6580, and n.s. p=0.9375 vs. *Tem1*$^{wt/wt}$, respectively. **(G)** Representative microscopic images of *in situ* zymography (with DAPI). The lung section (left panel) served as a positive control and the other co-incubated with EDTA (right panel) served as a negative control. **(H)** Representative microscopic images of DHE staining for reactive oxygen species generation. The lung section (left panel) served as a positive control and the other co-incubated with NAC (right panel) served as a negative control. (L indicates lumen. All scale bars represent 100 μm. Data are represented as mean values ± SEM.)

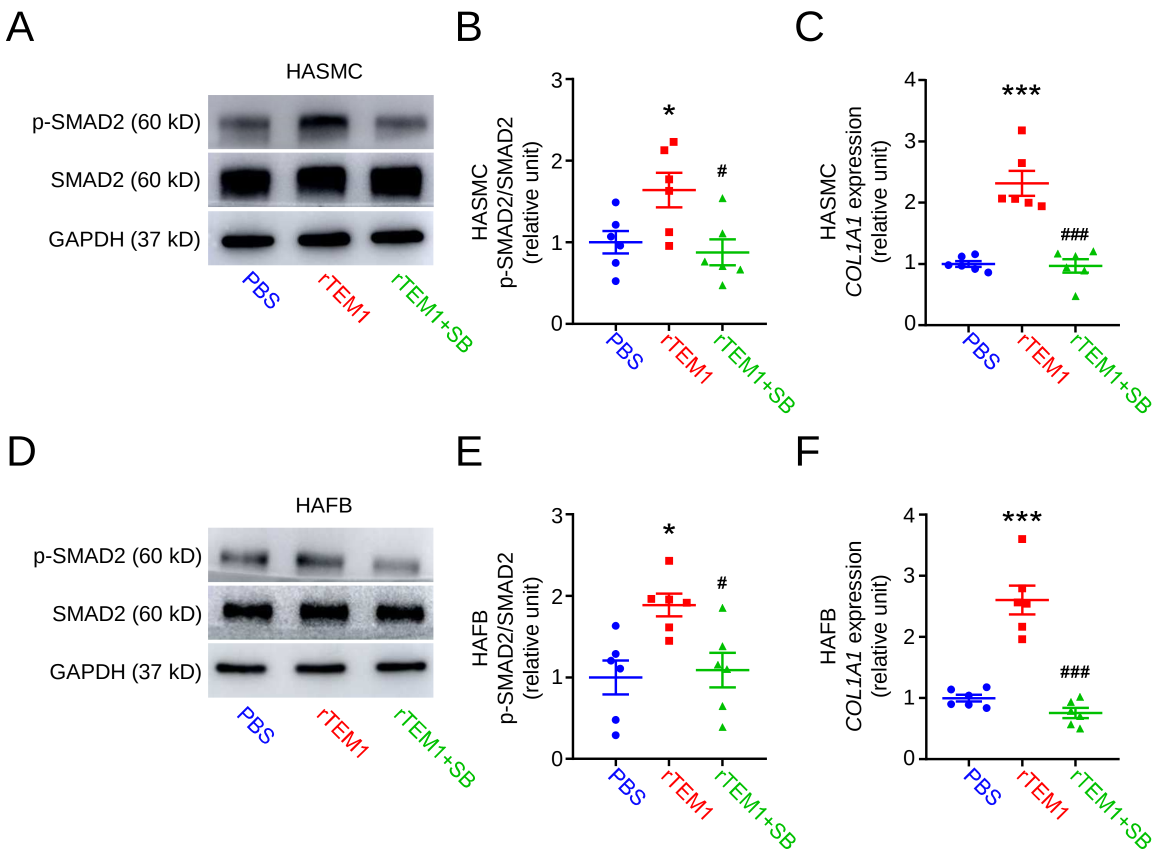

Fig. S7. SB-431542, a TGF-β receptor inhibitor, blocks rTEM1-induced SMAD2 phosphorylation and *COL1A1* gene expression in HASMCs and HAFBs. HASMCs (A-C) and HAFBs (D-F) were treated with PBS, 100 nM rTEM1, or 100 nM rTEM1 plus 10 μM SB-431542. **(A)** Representative western blot analysis of p-SMAD2 and SMAD2 levels at 15 min. **(B)** Quantification of p-SMAD2/SMAD2 levels (n=6). *p=0.0471 vs. PBS. #p=0.0175 vs. rTEM1. **(C)** *COL1A1* gene expression levels at 1 day (n=6). ***p<0.0001 vs. PBS. ###p<0.0001 vs. rTEM1. **(D)** Representative western blot analysis of p-SMAD2 and SMAD2 levels at 15 min. **(E)** Quantification of p-SMAD2/SMAD2 levels (n=6). *p=0.0125 vs. PBS. #p=0.0241 vs. rTEM1. **(F)** *COL1A1* gene expression levels at 1 day (n=6). ***p<0.0001 vs. PBS. ###p<0.0001 vs. rTEM1. (SB=SB-431542. Data are represented as mean values ± SEM.)

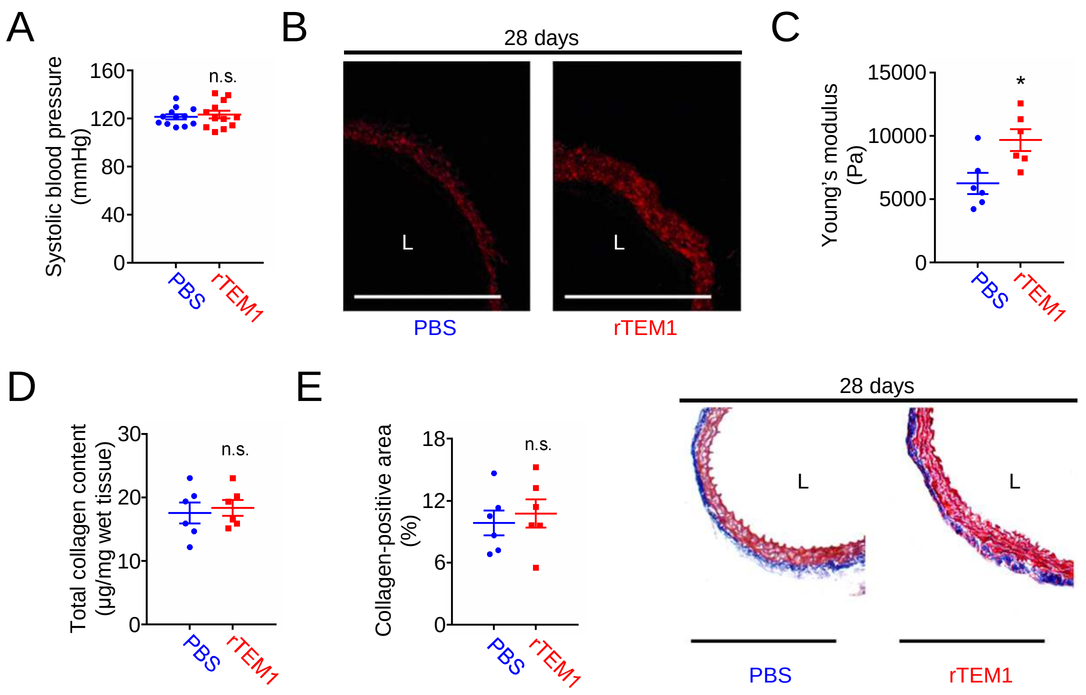

Fig. S8. Treatment with rTEM1 enhances aortic stiffness of the infrarenal aorta (the aneurysm site) but does not increase collagen content of the suprarenal aorta (the non-lesion site) in the $CaCl_2$-induced AAA model. (A) Systolic blood pressure at 28 days (n=12). n.s. p=0.6988 vs. PBS. **(B)** Representative microscopic images of picrosirius red staining. **(C)** AFM indentation results of aortic samples at 28 days (n=6). *p=0.0167 vs. PBS. **(D)** Total collagen content of the suprarenal aorta at 28 days (n=6). n.s. p=0.7070 vs. PBS. **(E)** Collagen-positive area of the suprarenal aorta at 28 days (n=6) and representative microscopic images of Masson's trichrome staining. n.s. p=0.6300 vs. PBS. (L indicates lumen. All scale bars represent 100 µm. Data are represented as mean values ± SEM.)

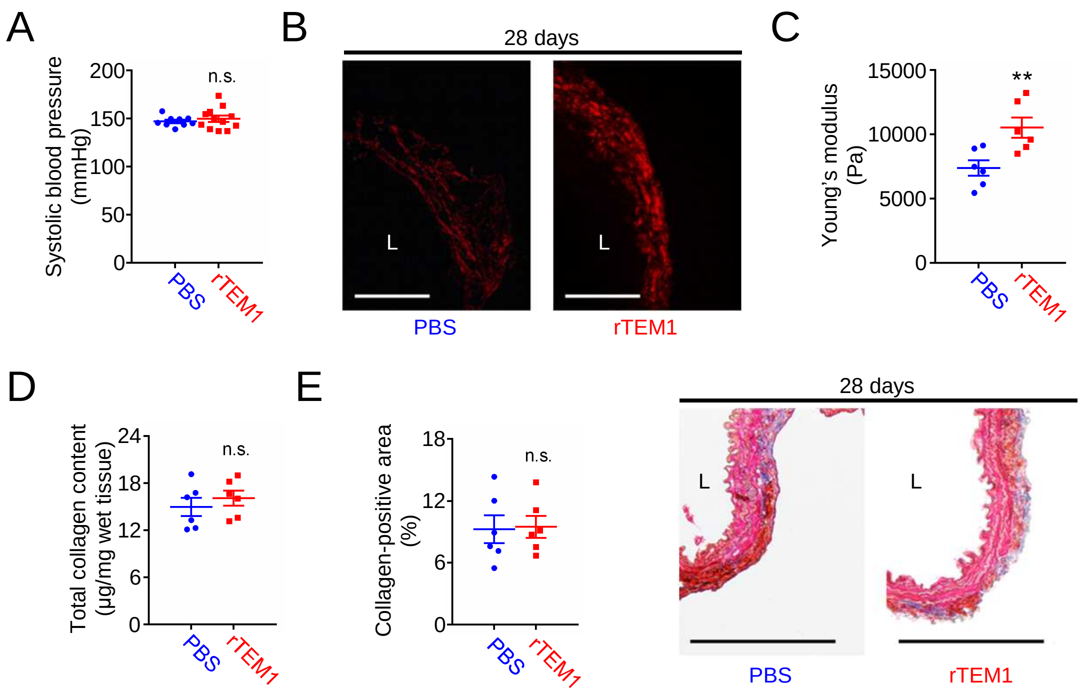

Fig. S9. Treatment with rTEM1 enhances aortic stiffness of the suprarenal aorta (the aneurysm site) but does not increase collagen content of the infrarenal aorta (the non-lesion site) in the AngII-infused AAA model. (A) Systolic blood pressure at 28 days (n=9 and 12). n.s. p=0.5143 vs. PBS. **(B)** Representative microscopic images of picrosirius red staining. **(C)** AFM indentation results of aortic samples at 28 days (n=6). **p=0.0099 vs. PBS. **(D)** Total collagen content of the infrarenal aorta at 28 days (n=6). n.s. p=0.4669 vs. PBS. **(E)** Collagen-positive area of the infrarenal aorta at 28 days (n=6) and representative microscopic images of Masson's trichrome staining. n.s. p=0.8921 vs. PBS. (L indicates lumen. All scale bars represent 50 µm. Data are represented as mean values ± SEM.)

Supplementary Methods

Supplementary Method 1. Seurat code used in this study.

```
#Library
library(Seurat)
library(dplyr)
library(ggplot2)
library(SingleR)

##Load the dataset
#sample1_sham
sample1_sham.data <- Read10X(data.dir = "C:/Users/user/Desktop/AAA_mouse2/sham")
sample1_sham <- CreateSeuratObject(counts = sample1_sham.data, project =
"sample1_sham", min.cells = 3, min.features = 10)
sample1_sham

#sample2_aaa
sample2_aaa.data <- Read10X(data.dir = "C:/Users/user/Desktop/AAA_mouse2/aaa")
sample2_aaa <- CreateSeuratObject(counts = sample2_aaa.data, project = "sample2_aaa",
min.cells = 3, min.features = 10)
sample2_aaa

##Quality control
#sham
#Add columns to object metadata.
sample1_sham$TN.merge <- "sample1_sham"
#The [[ operator can add columns to object metadata. This is a great place to stash QC stats
sample1_sham[["percent.mt"]] <- PercentageFeatureSet(sample1_sham, pattern = "^mt-")
#Show QC metrics for the first 5 cells
head(sample1_sham@meta.data, 5)
#Visualize QC metrics as a violin plot
VlnPlot(sample1_sham, features = c("nFeature_RNA", "nCount_RNA", "percent.mt"), ncol =
3)
#Filter out for Feature and mitochondria pct.
sample1_sham <- subset(sample1_sham, subset = nFeature_RNA > 200 & nFeature_RNA <
6000 & percent.mt < 10)

#aaa
#Add columns to object metadata.
sample2_aaa$TN.merge <- "sample2_aaa"
#The [[ operator can add columns to object metadata. This is a great place to stash QC stats
sample2_aaa[["percent.mt"]] <- PercentageFeatureSet(sample2_aaa, pattern = "^mt-")
#Show QC metrics for the first 5 cells
head(sample2_aaa@meta.data, 5)
#Visualize QC metrics as a violin plot
VlnPlot(sample2_aaa, features = c("nFeature_RNA", "nCount_RNA", "percent.mt"), ncol =
3)
#Filter out for Feature and mitochondria pct.
sample2_aaa <- subset(sample2_aaa, subset = nFeature_RNA > 200 & nFeature_RNA < 6000
```

```
& percent.mt < 10)

##Recheck
sample1_sham
sample2_aaa

##scRNA-seq sample integration
#Set merge file
TN.merge <- merge (sample1_sham, y = c(sample2_aaa), add.cell.ids =
c("sample1_sham","sample2_aaa"), project = "groups")
table(TN.merge$orig.ident)
#Set splitobject file
TN.list<- SplitObject(TN.merge, split.by = "orig.ident")
TN.list
TN.list <- lapply(X = TN.list, FUN = function(x) {
   x <- NormalizeData(x,normalization.method = "LogNormalize", scale.factor = 10000)
   x <- FindVariableFeatures(x, selection.method = "vst", nfeatures = 2000)})
#Select features that are repeatedly variable across datasets for integration
features <- SelectIntegrationFeatures(object.list = TN.list)
#Perform integration
reference.list <- TN.list[c("sample1_sham","sample2_aaa")]
TN.anchors <- FindIntegrationAnchors(object.list = reference.list, dims = 1:30)
#Create an 'integrated' data assay
TN.combined <- IntegrateData(anchorset = TN.anchors, dims = 1:30)
#Perform an integrated analysis
DefaultAssay(TN.combined) <- "integrated"
#Run the standard workflow for visualization and clustering
TN.combined <- ScaleData(TN.combined, verbose = TRUE)
TN.combined <- RunPCA(TN.combined, npcs = 50, verbose = TRUE)
VizDimLoadings(TN.combined, dims = 1:2)
ElbowPlot(TN.combined, ndims= 50)
# Cluster the cells
TN.combined <- FindNeighbors(TN.combined, reduction = "pca", dims = 1:41)
TN.combined <- FindClusters(TN.combined, resolution = 0.3)
table(TN.combined@active.ident)
table(Idents(TN.combined), TN.combined$orig.ident)
CellNumber <- table(Idents(TN.combined), TN.combined$orig.ident)
write.csv(CellNumber , file = "C:/Users/user/Desktop/CellNumber.csv")

##UMAP
TN.combined <- RunUMAP(TN.combined, reduction = "pca", dims = 1:41)
DimPlot(TN.combined, reduction = "umap",label = TRUE, pt.size = 0.8)


##Heatmap
Heatmapall <- subset(TN.combined, idents = c(0,1,2,3,4,5,6,7,8,9,10,11))
Heatmapall.markers <- FindAllMarkers(Heatmapall, only.pos = TRUE, min.pct = 0.25,
logfc.threshold = 0.5)
head(Heatmapall.markers)
write.csv(Heatmapall.markers , file = "C:/Users/user/Desktop/FindAllMarkers.csv",
```

```
col.names = TRUE)
top10 <- Heatmapall.markers %>% group_by(cluster) %>% top_n(n = 10, wt = avg_log2FC)
DoHeatmap(Heatmapall, features = top10$gene)

##Save the merged Seurat object
saveRDS(TN.combined, file = "C:/Users/user/Desktop/AAA_mouse2/aaa.combined.rds")
TN.combined <- readRDS(file = "C:/Users/user/Desktop/AAA_mouse2/aaa.combined.rds")

#Annotate cell types by SingleR
counts <- GetAssayData(TN.combined)
singler <- CreateSinglerObject(counts=counts, project.name = "TN", technology = "10X",
species = "Mouse", citation = "", ref.list = list(hpca=hpca), normalize.gene.length = FALSE,
variable.genes = "de", fine.tune = FALSE, reduce.file.size = TRUE, do.signatures = FALSE,
do.main.types = TRUE, numCores = SingleR.numCores)
for (ref.set in names(singler$singler) )
{
   types <- singler$singler[[ref.set]]$SingleR.single.main$labels[,1]
   cat("==== ", ref.set, ": ====\n")
   show(sort(table(types), decreasing=TRUE))
}
TN.combined[["rnaClusterID"]] <- Idents(TN.combined)
types <- singler$singler[[ref.set]]$SingleR.single.main$labels[,1]
clustering.table_hpca <- table(singler$singler[[ref.set]]$SingleR.single.main$labels[,1],
TN.combined$rnaClusterID)
clustering.table_hpca
write.csv(clustering.table_hpca , file = "C:/Users/user/Desktop/SingleR_hpca.csv")

singler <- CreateSinglerObject(counts=counts, project.name = "TN", technology = "10X",
species = "Mouse", citation = "", ref.list = list(bpe=blueprint_encode), normalize.gene.length
= FALSE, variable.genes = "de", fine.tune = FALSE, reduce.file.size = TRUE, do.signatures =
FALSE, do.main.types = TRUE, numCores = SingleR.numCores)
for (ref.set in names(singler$singler) )
{
   types <- singler$singler[[ref.set]]$SingleR.single.main$labels[,1]
   cat("==== ", ref.set, ": ====\n")
   show(sort(table(types), decreasing=TRUE))
}
TN.combined[["rnaClusterID"]] <- Idents(TN.combined)
types <- singler$singler[[ref.set]]$SingleR.single.main$labels[,1]
clustering.table_bpe <- table(singler$singler[[ref.set]]$SingleR.single.main$labels[,1],
TN.combined$rnaClusterID)
clustering.table_bpe
write.csv(clustering.table_bpe , file = "C:/Users/user/Desktop/SingleR_bpe.csv", col.names =
TRUE)

##Vlnplot
DefaultAssay(TN.combined) <- "RNA"
##Classical markers
VlnPlot(TN.combined, features = c("Cd248"),pt.size = FALSE, split.by = "orig.ident", cols=
c("#00BFC4","#F8766D"))
```

```
VlnPlot(TN.combined, features = c("Col1a1"),pt.size = FALSE, split.by = "orig.ident", cols=
c("#00BFC4","#F8766D"))

##Rename clusters
new.cluster.ids <- c("FB-1","Mono-1","SMC-1","FB-2","Mono-2","Neu",
                     "EC","Tcell","Mono-3","Bcell","DC","SMC-2")
names(new.cluster.ids) <- levels(TN.combined)
TNname.combined <- RenameIdents(TN.combined, new.cluster.ids)
DimPlot(TNname.combined, reduction = "umap",label = TRUE, pt.size = 0.8)
```

Supplementary Method 2. CellChat code used in this study.

```
#Library
library(Seurat)
library(CellChat)
library(ggplot2)
library(patchwork)
library(ggalluvial)
library(igraph)
library(dplyr)

TN.combined <- readRDS(file = "C:/Users/user/Desktop/AAA_mouse2/aaa.combined.rds")

##Rename cluster (for example：Fibroblast、Macrophage......)
new.cluster.ids <- c("FB-1","Mono-1","VSMC-1","FB-2","Mono-2","Neu",
                     "EC","Tcell","Mono-3","Bcell","DC","VSMC-2")
names(new.cluster.ids) <- levels(TN.combined)
TNname.combined <- RenameIdents(TN.combined, new.cluster.ids)
DimPlot(TNname.combined, reduction = "umap",label = FALSE, pt.size = 0.8)
DimPlot(TNname.combined, reduction = "umap",label = TRUE, pt.size = 0.8)

##Download all interaction pathways of CellChatDB
CellChatDB$interaction
write.csv(CellChatDB$interaction, file="C:/Users/user/Desktop/CellChatDB$interaction",
row.names=FALSE)

##Part I: Data input & processing and initialization of CellChat object
#Extract the CellChat input files from a Seurat V3 object
data.input <- GetAssayData(TNname.combined, assay = "RNA", slot = "data") # normalized
data matrix
labels <- Idents(TNname.combined)
meta <- data.frame(group = labels, row.names = names(labels)) # create a dataframe of the
cell labels

#Create a CellChat object using data matrix as input
cellchat <- createCellChat(object = data.input, meta = meta, group.by = "group")

#Add cell information into meta slot of the object
cellchat <- addMeta(cellchat, meta = meta, meta.name = "labels")
cellchat <- setIdent(cellchat, ident.use = "labels") # set "labels" as default cell identity
levels(cellchat@idents) # show factor levels of the cell labels
groupSize <- as.numeric(table(cellchat@idents)) # number of cells in each cell group

#Set the ligand-receptor interaction database
CellChatDB <- CellChatDB.mouse # use CellChatDB.mouse if running on mouse data
showDatabaseCategory(CellChatDB)

# Show the structure of the database
dplyr::glimpse(CellChatDB$interaction)
```

```
##Download all interaction pathways of CellChatDB
#CellChatDB$interaction
#write.csv(CellChatDB$interaction, file="C:/Users/user/Desktop/CellChatDB$interaction",
row.names=FALSE)

# use a subset of CellChatDB for cell-cell communication analysis
#CellChatDB.use <- subsetDB(CellChatDB, search = "") # use Secreted Signaling
# use all CellChatDB for cell-cell communication analysis
CellChatDB.use <- CellChatDB # simply use the default CellChatDB

# set the used database in the object
cellchat@DB <- CellChatDB.use

#Preprocessing the expression data for cell-cell communication analysis
cellchat <- subsetData(cellchat) # subset the expression data of signaling genes for saving
computation cost
future::plan("multisession", workers = 4) # do parallel
#> Warning: [ONE-TIME WARNING] Forked processing ('multicore') is disabled
#> in future (>= 1.13.0) when running R from RStudio, because it is
#> considered unstable. Because of this, plan("multicore") will fall
#> back to plan("sequential"), and plan("multiprocess") will fall back to
#> plan("multisession") - not plan("multicore") as in the past. For more details,
#> how to control forked processing or not, and how to silence this warning in
#> future R sessions, see ?future::supportsMulticore
cellchat <- identifyOverExpressedGenes(cellchat)
cellchat <- identifyOverExpressedInteractions(cellchat)
cellchat <- projectData(cellchat, PPI.mouse)

##Part II: Inference of cell-cell communication network
#Compute the communication probability and infer cellular communication network
cellchat <- computeCommunProb(cellchat, raw.use = TRUE)
# Filter out the cell-cell communication if there are only few number of cells in certain cell
groups
cellchat <- filterCommunication(cellchat, min.cells = 5)

#Extract the inferred cellular communication network as a data frame
df.net <- subsetCommunication(cellchat)

#Infer the cell-cell communication at a signaling pathway level
cellchat <- computeCommunProbPathway(cellchat)

#Calculate the aggregated cell-cell communication network
cellchat <- aggregateNet(cellchat)

##Part III: Visualization of cell-cell communication network
pathways.show <- c("TGFb")

# Hierarchy plot
vertex.receiver = seq(1,6) # a numeric vector.
netVisual_aggregate(cellchat, signaling = pathways.show, layout = "hierarchy",
```

```
vertex.receiver = vertex.receiver)

#Compute the contribution of each ligand-receptor pair to the overall signaling pathway and
visualize cell-cell communication mediated by a single ligand-receptor pair
netAnalysis_contribution(cellchat, signaling = pathways.show)

# Compute the network centrality scores
cellchat <- netAnalysis_computeCentrality(cellchat, slot.name = "netP") # the slot 'netP'
means the inferred intercellular communication network of signaling pathways
# Visualize the computed centrality scores using heatmap, allowing ready identification of
major signaling roles of cell groups
netAnalysis_signalingRole_network(cellchat, signaling = pathways.show, width = 8, height =
2.5, font.size = 10)

#Visualize the dominant senders (sources) and receivers (targets) in a 2D space
#> Signaling role analysis on the aggregated cell-cell communication network from all
signaling pathways
# Signaling role analysis on the cell-cell communication networks of interest
netAnalysis_signalingRole_scatter(cellchat, signaling = pathways.show, label.size = 6)

##Save the CellChat object
saveRDS(cellchat, file = "C:/Users/user/Desktop/AAA_mouse/cellchat_all.rds")
```
